# Global analysis of the yeast knockout phenome

**DOI:** 10.1126/sciadv.adg5702

**Published:** 2023-05-26

**Authors:** Gina Turco, Christie Chang, Rebecca Y. Wang, Griffin Kim, Emily H. Stoops, Brianna Richardson, Vanessa Sochat, Jennifer Rust, Rose Oughtred, Nathaniel Thayer, Fan Kang, Michael S. Livstone, Sven Heinicke, Mark Schroeder, Kara J. Dolinski, David Botstein, Anastasia Baryshnikova

**Affiliations:** ^1^ Calico Life Sciences LLC, South San Francisco, CA, USA.; ^2^ Lewis-Sigler Institute for Integrative Genomics, Princeton University, Princeton, NJ, USA.; ^3^ Lawrence Livermore National Laboratory, Livermore, CA, USA.

## Abstract

Genome-wide phenotypic screens in the budding yeast *Saccharomyces cerevisiae*, enabled by its knockout collection, have produced the largest, richest, and most systematic phenotypic description of any organism. However, integrative analyses of this rich data source have been virtually impossible because of the lack of a central data repository and consistent metadata annotations. Here, we describe the aggregation, harmonization, and analysis of ~14,500 yeast knockout screens, which we call Yeast Phenome. Using this unique dataset, we characterized two unknown genes (*YHR045W* and *YGL117W*) and showed that tryptophan starvation is a by-product of many chemical treatments. Furthermore, we uncovered an exponential relationship between phenotypic similarity and intergenic distance, which suggests that gene positions in both yeast and human genomes are optimized for function.

## Teaser

The harmonization of ~14,500 knockout screens gives insight into the functional organization of yeast and human genomes.

## INTRODUCTION

Connecting genotypes to phenotypes is essential for understanding the molecular architecture of complex traits and developing successful therapies against aging and disease. The assembly of large human cohorts, coupled with deep phenotyping and advanced computational analysis, is enabling great progress toward uncovering genome-wide phenotypic associations in natural human populations (*1*). However, inferring causal gene-trait relationships from these associations remains a challenge because of the complexity of human genetics, physiology, socioeconomic structure, and environmental exposures. An orthogonal approach to map genes to phenotypes has long been available through model organisms that allow systematic gene-by-gene perturbations in isogenic backgrounds and carefully controlled experimental environments.

The budding yeast *Saccharomyces cerevisiae* has pioneered the systematic phenotypic analysis of gene perturbations (*2*). In 2002, a consortium of laboratories released the yeast knockout (YKO) collection, which provided a complete set of isogenic strains each deleted for exactly one open reading frame (ORF) (*3*). This collection, along with progress in automation and parallelization, enabled rapid, affordable, and comprehensive loss-of-function screens that examined nearly every aspect of yeast biology that could be measured on a large scale. However, the results of these screens remained physically scattered and disorganized, thus preventing systematic analysis and integration. In the absence of a central repository and consistent metadata annotations, it has been impossible to know exactly which experiments have been done, how they compare to one another, and what information they contribute to our global understanding of yeast as a complex biological system. Here, we address this problem and describe Yeast Phenome, a data library that aggregates and annotates all published screens of the YKO collection. Currently, Yeast Phenome contains ~43 million causal gene-to-phenotype links, which represent the largest, richest, and most systematic phenotypic description of any organism. To encourage exploration, download, and analysis, we have made all data and metadata available at www.yeastphenome.org.

The aggregation and harmonization of YKO data in Yeast Phenome provide a unique dataset and enable discoveries that could not be made with any single experiment in isolation. To demonstrate its value, we provide several examples of Yeast Phenome data analysis and describe three key findings, ranging from gene-level to system-level observations. First, we analyze the variation in the number of phenotypes per gene and find that tryptophan biosynthesis is an exceptional metabolic pathway that is required for resistance to more than 1000 chemical compounds. Second, we show that a multidimensional phenotypic profile, i.e., the set of all known phenotypes associated with a gene, is a strong predictor of gene function that complements and reinforces other genomic datasets. Using phenotypic profiles as predictive tools, we identify and validate the roles of two uncharacterized ORFs (*YHR045W* and *YGL117W*). Last, we uncover an unexpected relationship between phenotypic profile similarity and intergenic distance, which potentially reflects the functional architecture of yeast and human genomes. Overall, we show that data curation is a powerful approach for generating new datasets and identifying global patterns that are not apparent on a smaller scale.

## RESULTS

### Building a data library of knockout phenotypic screens

**Fig. 1.**
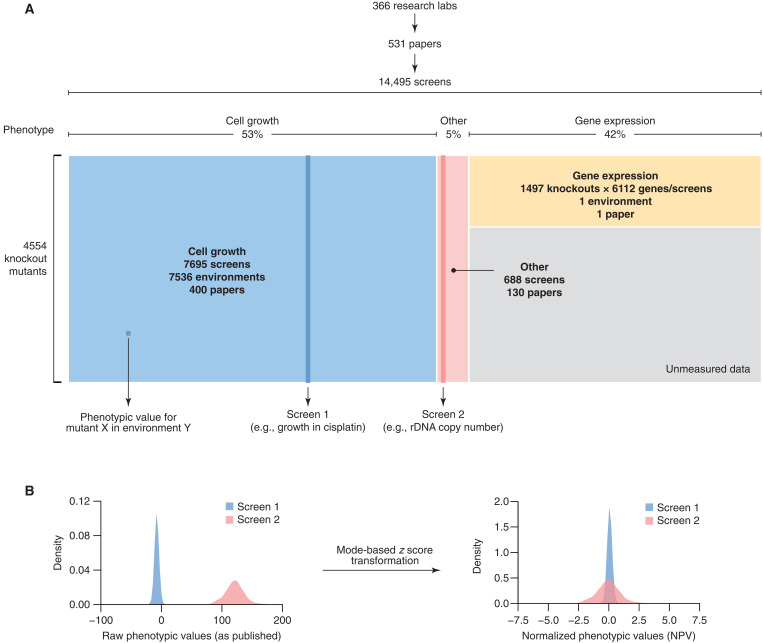
Yeast Phenome is a data library of published genome-scale screens of the YKO collection. (**A**) Yeast Phenome (www.yeastphenome.org) can be thought of as a data matrix where each row is a knockout mutant and each column is a phenotypic screen. The matrix contains phenotypic values obtained by extracting data from 531 papers published by 366 research laboratories. The phenotypes tested by the screens and the experimental conditions/environments, in which the phenotypes were tested (e.g., chemical compounds, pH, temperature, and growth media), were annotated using standard vocabularies. Three major classes of phenotypes (cell growth, gene expression, and other) are highlighted in blue, yellow, and pink, respectively. Gray represents unmeasured data because gene expression profiles were tested for only ~1500 knockout mutants. (**B**) To facilitate analysis and interpretation, raw phenotypic values (i.e., those released in the publication) were normalized using a modified *z* score transformation that uses the mode (instead of the mean) and SD from the mode to shift and scale the data.

The YKO is a collection of ~5000 yeast strains where every ORF is individually deleted and replaced by a selectable marker linked to an ORF-specific molecular barcode in a common genetic background (fig. S1, A and B). An exhaustive survey of the literature (Materials and Methods) showed that, between November 2000 and May 2022, 366 research groups published 531 studies, each describing the systematic testing of at least 1000 haploid or homozygous diploid YKO mutants for one or more phenotypes under one or more experimental conditions (Fig. 1A). To examine the wealth of information concealed in these data, we curated the 531 publications and assembled a comprehensive compendium of 14,495 knockout screens (Fig. 1A). We developed standard vocabularies to annotate and cross-reference 6731 phenotypes and 7536 experimental environments associated with the screens and devised a reproducible computational pipeline for extracting, formatting, and normalizing data from each publication (fig. S1C and Materials and Methods). Through close collaboration with 150 yeast researchers, we recovered extended data for 413 screens (3% of the total) that correspond to more complete and, typically, more quantitative versions of previously published experiments (Materials and Methods).

The Yeast Phenome data library is experimentally and biologically diverse (Fig. 1A). The most tested phenotype (53% of all screens) is cell growth measured by colony size, optical density in liquid culture, relative barcode abundance in pools, and several other metrics (Fig. 1A, blue). Because it is relatively easy to measure, growth of ~5000 knockout mutants has been tested in 7536 different environments, most of which (96%) involved a chemical compound of known or unknown mode of action (Fig. 1A). For ~1500 knockout mutants (~30% of the YKO collection), growth measurements are supplemented by mRNA expression levels of 6112 genes, representing the second most common phenotype in Yeast Phenome (42% of all screens; Fig. 1A, yellow). Despite being measured primarily in a single unperturbed environment (*4*), these genome-wide expression profiles of knockout mutants provide a large and diverse set of molecular biomarkers that may act upstream of other phenotypes, including response to chemical treatments. The remaining 5% of screens in Yeast Phenome are a mosaic of 670 phenotypes that describe the state of the genome, proteome, and metabolome of knockout mutants, along with morphological parameters and other cellular phenotypes, such as protein localization and intracellular pH (Fig. 1A, red). These phenotypes are generally measured using advanced technologies (e.g., mass spectrometry, next-generation sequencing, and high-resolution microscopy), complex reporter systems, and, sometimes, longitudinal sampling, which probe yeast biology in greater detail but are limited in throughput (on average, 5.7 screens per publication). Hence, they create many small but valuable datasets that are scattered throughout the literature and have never been examined in the context of other datasets.

To facilitate the analysis and interpretation of diverse Yeast Phenome data, we implemented several conventions and normalizations (Materials and Methods). Because different phenotypes followed markedly different distributions but were consistently unimodal, we used the mode as a reference to normalize each screen using a modified *z* score transformation (Fig. 1B and Materials and Methods). As a result, all phenotypic values reported in Yeast Phenome can be universally interpreted as standardized deviations from the most typical mutant, which, assuming that extreme phenotypes are rare, is also likely to resemble the wild-type strain. Both original and transformed data, which we refer to as normalized phenotypic values (NPVs), are available at www.yeastphenome.org.

### Yeast Phenome data are reproducible and provide unique information about gene function

Because of its size and metadata annotations, Yeast Phenome provides an opportunity to investigate the quality of YKO data and to test their robustness to common sources of experimental noise. For example, we can easily identify and compare independent screens that examined the same phenotype under similar experimental conditions and therefore assess the biological reproducibility of the phenotype. To demonstrate this point, we compared eight independent screens of respiratory metabolism (i.e., growth on glycerol) and found that, on average, 71% of respiration-deficient mutants (NPV < −3) identified in any one screen were reproduced in at least five of the eight replicates (note S1 and fig. S2A). The reproducibility of respiration deficiency across the eight screens was nearly complete (cosine ρ = 0.994 ± 0.003, mean ± SD) when, instead of a gene-by-gene overlap, we compared the phenotype’s enrichment profiles across the genetic interaction similarity network using Spatial Analysis of Functional Enrichment (SAFE) (note S1 and fig. S2B) (*5*).

To examine reproducibility across a wider range of phenotypes and conditions, we identified 164 pairs of near-replicate screens and compared their NPVs, regardless of significance (Materials and Methods). We found that 30% of near-replicate screen pairs have similar phenotypic profiles (cosine ρ > 0.6) and 48% have similar SAFE enrichment profiles (ρ > 0.6), a 345-fold and 42-fold increase over background, respectively (fig. S3, A and B). Replicate screens performed by the same laboratory (as inferred from the name of the last author of the corresponding publication) were more similar than screens performed by different laboratories (68% versus 39% of screen pairs with ρ > 0.6; fig. S3B), likely reflecting differences in YKO versions, housing conditions, phenotyping strategies, and other experimental parameters that are not easily captured.

Another potential source of experimental noise in YKO data is secondary mutations (i.e., “suppressors”) that arise spontaneously as adaptations to gene loss and may interfere with the correct assignment of genes to functions. To measure the impact of such strain evolution, we compared different versions of the YKO collection, as well as strains with and without evidence of secondary mutations (note S2 and fig. S4). We found that secondary mutations increase the relative risk of incorrect gene-to-function assignment by no more than 3% and, therefore, are unlikely to impede the use and interpretation of YKO data (note S2).

High-quality knockout phenotypes provide strong experimental evidence of gene function and have long been exploited to identify key players in major biological pathways. A multidimensional phenotypic profile, i.e., a vector of binary or quantitative phenotypic values associated with a given gene, is even more powerful at predicting gene function because it enables more robust comparisons of known and unknown genes, and facilitates transfers of knowledge through “guilt by association.” We asked how well gene function can be predicted by phenotypic profiles assembled in Yeast Phenome relative to other sources of functional information, such as gene expression, genetic interactions, and protein-protein interactions (Materials and Methods). In each dataset, we ranked all gene pairs by their profile similarity and performed a precision-recall analysis using membership in the same functional group [a protein complex, a biochemical pathway, or a moderately specific Gene Ontology (GO) biological process term] as ground truth for a functional relationship (Materials and Methods). We found that profile similarity in each dataset is comparably predictive of a functional relationship [area under the precision-recall curve (AUPR) = 0.424 to 0.477; fig. S5A]. However, different types of functional relationships are better predicted by different types of biological data (fig. S5B). For example, genes acting in the same biochemical pathway are best predicted by coexpression profiles (AUPR = 0.258), whereas shared membership in the same protein complex is best predicted by similar knockout phenotypes (AUPR = 0.429). Despite a consistent performance overall, we observed little redundancy between data types such that genes correlated in one dataset were largely uncorrelated in others (fig. S5C). We conclude that each data type provides independent functional information that should be regarded as complementary and analyzed in an integrative manner.

Given the diversity of data in Yeast Phenome, we also asked how many YKO screens contribute independent functional information (Materials and Methods). We found that 50% of phenotypic variation among knockout mutants is explained by 273 principal components (fig. S6A), confirming our intuitive expectation that many screens are independent and complementary. The main axis of variation, which accounts for ~4% of the total variance, separates genes involved in protein synthesis from those involved in vesicular transport (fig. S6B). These genes are resistant and sensitive to an exceptionally wide range of exogenous stresses and have been proposed to act as key regulators of cellular homeostasis (*3*, *6*–*8*). The second axis of variation shows a significant correlation to mutant growth rate (Pearson *R* = 0.21, *P* = 10^−44^; fig. S6B), another important factor in general stress resistance and other phenotypes (*4*, *8*–*10*).

### Phenotype rates vary across genes and biological processes

**Fig. 2.**
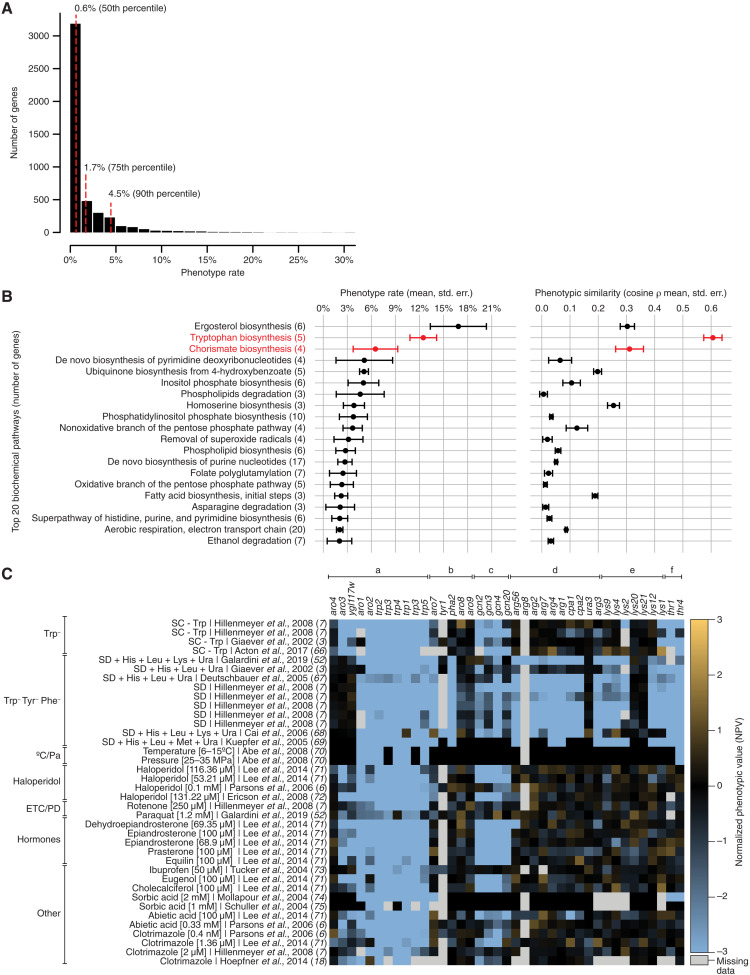
Tryptophan biosynthesis is essential for resistance to a wide range of chemical compounds. (**A**) Distribution of phenotype rates for all genes in Yeast Phenome. (**B**) The biosynthesis of tryptophan and its precursor chorismate are two of the top three biochemical pathways with the highest phenotype rate and the highest phenotypic similarity. Given a list of genes encoding members of a biochemical pathway, we computed the mean and SE (std. err.) of their phenotype rates, as well as the mean and SE for their pairwise phenotypic similarities. Of 187 tested pathways, the 20 pathways with the highest mean phenotype rates are shown. Tryptophan and chorismate biosynthesis are highlighted in red. The number of genes in each pathway is indicated in parentheses. (**C**) Mutants involved in the biosynthesis of tryptophan (*trp1*–*5*) and chorismate (*aro1*–*4*), but not other amino acids, share sensitivity to tryptophan-depleted media, low temperature, high pressure, and a wide range of chemical compounds. The heatmap shows NPVs for a set of mutants (columns) in a sample of screens (rows). Mutants (columns) are organized by pathway and include the following: (a) biosynthesis of chorismate and tryptophan, (b) biosynthesis of tyrosine and phenylalanine, (c) the general amino acid control (GAAC) pathway, (d) biosynthesis of arginine, (e) biosynthesis of lysine, and (f) biosynthesis of threonine. Screens (rows) are organized by tested condition and include growth in the following: tryptophan-limited media (Trp^−^); media limited for multiple amino acids, including tryptophan, tyrosine, and phenylalanine (Trp^−^ Tyr^−^ Phe^−^); exposure to low temperature and high pressure (°C/Pa); exposure to haloperidol (Haloperidol); exposure to rotenone and paraquat (ETC/PD); exposure to human hormones (Hormones); and exposure to other chemical compounds (Other).

NPVs, which express a mutant’s phenotype as a standardized deviation from the most typical mutant in the corresponding phenotypic screen, allow us to compare phenotypes across different experiments and identify genes having the greatest impact on cell physiology. We found that virtually all genes have at least one strong phenotype in Yeast Phenome (|NPV| > 3), supporting earlier predictions that no gene is truly dispensable (*7*). Despite this common baseline, the gene-specific phenotype rate, defined as the fraction of screens in which a gene shows a strong phenotype (|NPV| > 3), is highly variable, ranging from ~0 to 31% (mean = 1.8%, median = 0.6%; Fig. 2A). As expected, genes with many phenotypes (top decile, phenotype rate > 4.5%) are more likely to lack a paralogue [odds ratio (OR) = 2.9, *P* = 6 × 10^−10^], to be conserved in higher organisms (OR = 2.7, *P* = 3.8 × 10^−13^), and to be annotated to multiple biological processes (OR = 7.4, *P* = 4.6 × 10^−33^) than genes with few phenotypes (bottom decile, phenotype rate < 0.3%). Phenotype rate is also not uniformly distributed across biological processes: Genes involved in intracellular membrane trafficking (e.g., intra-Golgi, Golgi-to-endosome, and Golgi-to-vacuole transport), pH regulation (e.g., vacuole organization and acidification), lipid metabolism (e.g., ergosterol biosynthesis), transcription, and chromatin remodeling have more phenotypes than expected by random chance (fig. S7A). In contrast, metabolic functions (e.g., transmembrane transport and metabolism of carbohydrates, metal ions, and nitrogen compounds) are generally depleted for phenotypes (fig. S7B).

In principle, differences in phenotype rate among biological processes could be caused by an ascertainment bias such that some phenotypes are over- or underrepresented in Yeast Phenome for technical, biological, or historical reasons. Alternatively, phenotype rates may vary because of differences in mutational robustness that arise from the presence or absence of compensatory mechanisms, masking the loss of a gene or a pathway. To evaluate the relative impact of ascertainment bias and mutational robustness, we compared phenotype rates to genetic interaction degrees for the same processes. We reasoned that, while phenotypic profiles may be subject to sampling bias, genome-wide genetic interaction profiles are virtually complete and unbiased (*11*). We found that the average phenotype rate and genetic interaction degree of a biological process are strongly correlated (Spearman ρ = 0.63, *P* = 6.4 × 10^−123^; fig. S8), suggesting that mutational robustness is an important contributor to phenotype rate. However, some processes, especially those related to vesicular trafficking and transcriptional regulation, display higher phenotype rates than predicted from their genetic interaction degrees (fig. S8). Such an excess of phenotypes suggests that vesicle transport and transcription were probed more frequently than other processes, either because researchers designed more screens to target these processes or because, unbeknownst to the researchers, these processes are required under more screening conditions.

### Tryptophan biosynthesis is essential for resistance to many chemical perturbations

Although knockout mutants of most metabolic genes have few phenotypes, we found that biosynthesis of aromatic amino acids is a notable exception and presents one of the highest phenotype rates of all biological processes (fig. S7A). The three aromatic amino acids (tryptophan, tyrosine, and phenylalanine) are synthesized from a common precursor, chorismate, via three separate pathways (fig. S9A). However, only genes involved in the biosynthesis of chorismate (*ARO1* to *ARO4*) and tryptophan (*TRP1* to *TRP5*) have high phenotype rates (on average, 6.5 and 12.5%, respectively, a 3.8- to 7.3-fold increase over the mean of all genes; Fig. 2B), whereas tyrosine and phenylalanine biosynthesis genes (*ARO7* to *ARO9*, *TYR1*, and *PHA2*) are close to average (1.7%). The phenotype rates of *trp*∆ and *aro*∆ mutants are the second and third highest among 187 biochemical pathways encoded in the yeast genome, following only ergosterol biosynthesis (Fig. 2B). Furthermore, *trp*∆ and *aro*∆ phenotypic profiles are the most highly correlated (cosine ρ = 0.60 ± 0.15 for *trp*∆ mutants, mean ± SD; *n* = 10 pairs; Fig. 2B), indicating that their phenotypes are likely biologically meaningful and not caused by experimental noise.

As expected, *trp*∆/*aro*∆ mutants share phenotypes such as the inability to grow on tryptophan-limited media (Fig. 2C, Trp^−^ and Trp^−^ Tyr^−^ Phe^−^), at low temperature or under high hydrostatic pressure (Fig. 2C, °C/Pa). Both of these latter conditions are associated with the down-regulation of the main tryptophan permease Tat2 and consequent repression of tryptophan uptake (*12*). The vast majority (99%) of *trp*∆ and *aro*∆ phenotypes are sensitivities to 1138 chemical compounds (NPV < −2), consistent with prior identification of *TRP1* to *TRP5*, *ARO1* and *ARO2* as multidrug resistance genes (*7*). The sensitivity of *trp*∆/*aro*∆ mutants suggests that these 1138 compounds modulate tryptophan uptake or metabolism through a direct or indirect mechanism (Discussion).

Although many compounds causing *trp*∆/*aro*∆ sensitivity are not easily identifiable because they are proprietary and lack a publicly available name or chemical structure (Materials and Methods), others are well-known chemicals with extensive evidence for a role in tryptophan homeostasis in yeast, rats, and other organisms. Examples of such known chemicals are haloperidol, rotenone, and paraquat. Haloperidol is an antipsychotic medication prescribed for the treatment of schizophrenia, Tourette syndrome, bipolar disorder, and substance abuse. Long-term haloperidol usage can cause patients to develop tardive dyskinesia (TD), a syndrome of involuntary repetitive body movements such as twitching, shaking, and grimacing (*13*). Such movements are greatly reduced by the dietary supplementation of tryptophan in haloperidol-induced rat models of TD (*14*). Rotenone and paraquat are broad-spectrum pesticides that target the electron transfer chain (ETC) and cause oxidative damage. Chronic exposure to both chemicals has been linked to the development of Parkinson’s disease (PD) in mice, rats, and humans (*15*). In a manner similar to haloperidol, dietary tryptophan improves the impaired motor functions in rotenone-induced rat models of PD (*16*). The benefits of tryptophan in animals exposed to haloperidol, rotenone, and paraquat, along with the sensitivity of yeast *trp*∆/*aro*∆ mutants to all three compounds (Fig. 2C, haloperidol and ETC/PD), lead us to speculate that these and, potentially, many other *trp*∆/*aro*∆ chemicals limit the availability of tryptophan in the human nervous system.

Environmental conditions and chemicals causing *trp*∆/*aro*∆ sensitivity do not affect the growth of other mutants defective in amino acid biosynthesis (e.g., arginine, lysine, threonine; Fig. 2C, d to f). These treatments therefore appear to specifically mimic tryptophan depletion, rather than a general state of amino acid starvation. In wild-type yeast, the availability of all amino acids, including tryptophan, is monitored by the general amino acid control (GAAC) pathway, which senses the accumulation of uncharged tRNAs and up-regulates the expression of biosynthetic genes (*17*). GAAC mutants (*gcn2*∆, *gcn3∆*, *gcn4∆*, and *gcn20∆*) are sensitive to only 38% of the conditions that cause *trp*∆/*aro*∆ sensitivity (Fig. 2C and fig. S9B), suggesting that, under these conditions, the concentration of tryptophanyl-tRNA molecules is decreased and GAAC is required to activate a proper response. In the remaining 62% of *trp*∆/*aro*∆ conditions, a functional GAAC is not required for survival, suggesting that tRNA charging is not affected and other tryptophan-derived molecules may be limiting (Discussion).

Overall, the tryptophan biosynthesis pathway appears to be uniquely important for resistance to a wide variety of chemical stresses, some of which may result in decreased tRNA charging. While the specific mechanism for these effects remains unknown, we speculate that *trp*∆*/aro*∆ compounds may disrupt the composition and/or fluidity of the plasma membrane, therefore affecting the function of membrane-bound tryptophan permeases (Discussion).

### Phenotypic profiles organize genes into functional domains

**Fig. 3.**
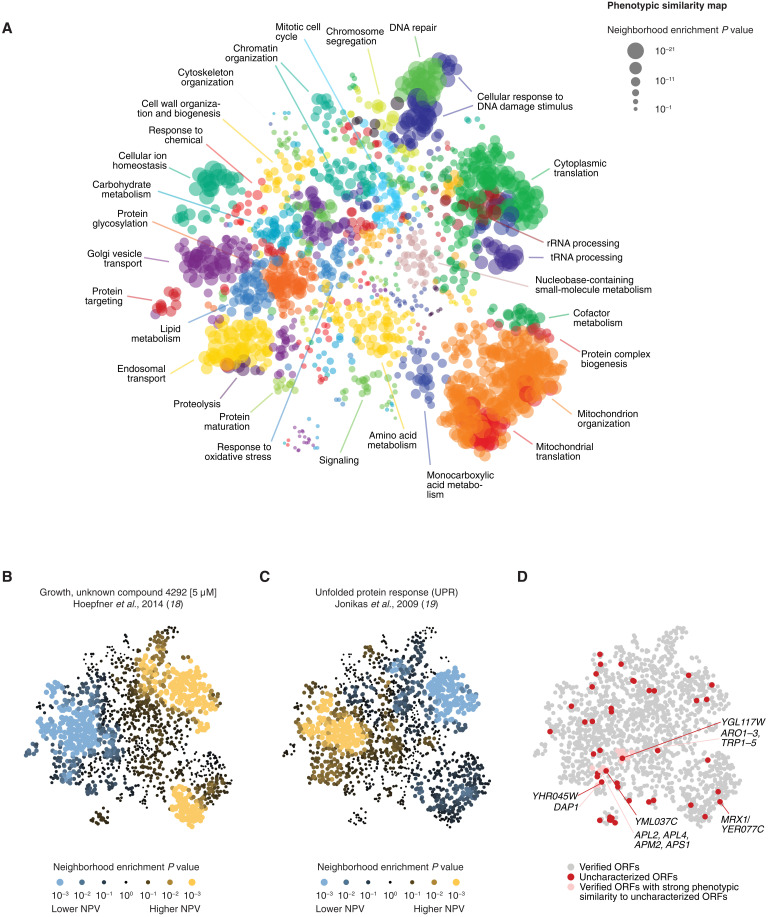
Phenotypic profiles organize genes by function, help interpret screen results, and validate uncharacterized ORFs. (**A**) A phenotypic similarity map was generated by applying Uniform Manifold Aapproximation and Projection (UMAP) to the phenotypic profiles of 1586 genes with a phenotype rate of >1%. The map, where genes with similar phenotypes are placed closer than genes with dissimilar phenotypes, was annotated using SAFE with GO Slim biological process terms. Nodes (genes) are colored on the basis of the GO term with the highest enrichment in their local neighborhoods. The regions with the strongest enrichments are labeled with the corresponding GO terms. (**B** and **C**) SAFE was used to annotate the map with NPVs from a chemical genomic screen of the unknown chemical compound 4292 (B) and a reporter screen for the unfolded protein response (UPR) (C). Nodes (genes) are colored on the basis of the average NPV in their local neighborhood relative to random expectation. (**D**) The phenotypic similarity map shows the distribution of uncharacterized ORFs and suggests hypotheses about their potential functions. Red nodes correspond to 43 uncharacterized ORFs (phenotype rate > 1%, similarity to a verified ORF ρ > 0.17). Pink nodes correspond to verified ORFs with strong phenotypic similarity to uncharacterized ORFs.

As shown above, phenotypic profiles are powerful tools for identifying functionally similar genes and transfer knowledge through guilt by association (fig. S5). To gain a global view of gene-gene relationships uncovered by phenotypic similarity, we selected 1586 genes, showing a strong phenotype (|NPV| > 3) in at least 1% of screens and projected the genes on a two-dimensional (2D) plane such that their relative distances reflected their phenotypic similarities (Fig. 3A and Materials and Methods). The resulting phenotypic similarity map, annotated with SAFE (*5*), showed that, similar to pairwise genetic interactions (*9*, *11*), knockout phenotypes organize genes into distinct yet closely connected domains, each enriched for one or more biological processes (Fig. 3A and Materials and Methods).

The phenotypic similarity not only map groups of genes in a way that reflects their shared function but also provides a key for interpreting new or poorly understood phenotypes. For example, the map can be annotated with the chemogenomic profile of an unknown compound to determine which biological processes are required for sensitivity or resistance to the chemical (Fig. 3B). SAFE analysis of one such compound, number 4292 in (*18*), shows that mutants in protein glycosylation, sorting and degradation pathways are sensitive to the chemical, whereas mutants in cytoplasmic and mitochondrial translation are relatively resistant (Fig. 3B). The SAFE enrichment profile of compound 4292 is a near mirror image of a fluorescent reporter-based screen for unfolded protein response (UPR) (Fig. 3C) that measures the activation of Hac1-regulated genes in response to the accumulation of misfolded proteins in the endoplasmic reticulum (*19*). While the name, molecular target, or chemical structure of compound 4292 is not publicly available (Materials and Methods), the reverse similarity of its SAFE profile to UPR (Pearson *R* = −0.87, *P* ~ 0) strongly suggests that compound 4292 impairs protein folding or quality control. Overall, the SAFE profiles of 1578 chemogenomic screens (21% of all tested chemicals) show a strong correlation (|*R*| > 0.7) to at least one nonchemogenomic phenotype. In addition to UPR, the phenotypes with the highest number of chemogenomic associations include vacuolar morphology; copy number and expression of mitochondrial DNA; chronological life span in glucose-limiting conditions; and intracellular concentrations of potassium, calcium, and glycogen (table S3).

### Phenotypic profiles enable annotation of uncharacterized ORFs

The ability of phenotypic profiles to organize genes by function provides an opportunity to validate uncharacterized ORFs and assign gene functions. The *Saccharomyces* Genome Database estimates that 688 yeast ORFs (10% of the genome) are currently uncharacterized, meaning that they are likely to produce a protein, as suggested by their conservation in other species, but no such protein product has been experimentally verified in *S. cerevisiae* yet (*20*). Of the 688 uncharacterized ORFs, 527 ORFs (77%) have at least 10 strong phenotypes in Yeast Phenome (|NPV| > 3) and 46 have robust phenotypic profiles that are predictive of function (phenotype rate > 1%, similarity to a verified ORF ρ > 0.17; Fig. 3D and table S4). We found that the top similarities of the uncharacterized ORFs and their positioning on the phenotypic similarity map are highly consistent with preliminary evidence from independent high-throughput experiments, whenever such evidence is available in the literature. For example, *MRX1*/*YER077C*, which appears to encode a protein localized to mitochondria (*21*) and interacting with the mitochondrial organization of gene expression complexes (*22*), is most similar to members of the mitochondrial translation machinery and localizes on the map accordingly (Fig. 3D). Another uncharacterized ORF, *YML037C*, maps next to *APL2*, *APL4*, *APM2*, and *APS1* as well as other members of the adaptor protein 1 (AP-1) clathrin-associated complex (Fig. 3D). This map position is consistent with fluorescence microscopy experiments showing that *YML037C* colocalizes with clathrin-coated vesicles (*21*). To encourage functional annotations of these and other uncharacterized ORFs, as well as verified ORFs without a known function, the Yeast Phenome website provides a set of tools to explore shared phenotypes, verify the mutants’ genomic sequences, and connect to the wealth of information available in other databases (www.yeastphenome.org). As a demonstration of the predictive power of phenotypic similarity, we closely examined two of the uncharacterized ORFs with the highest phenotypic similarity to a verified ORF and tested their predicted functions experimentally.

**Fig. 4.**
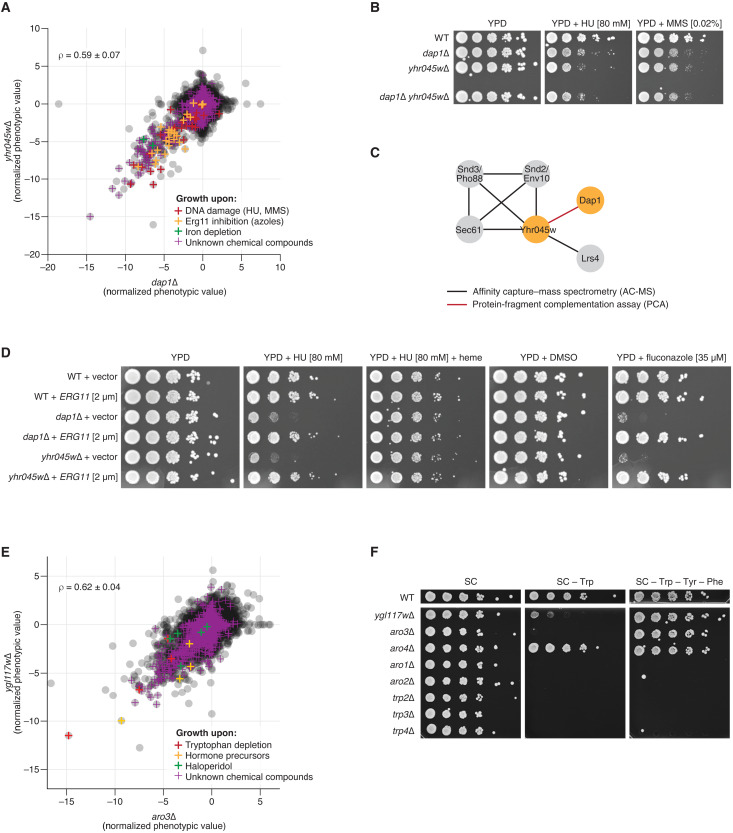
Functional validation of *YHR045W* and *YGL117W*. (**A**) The similarity of the phenotypic profiles of *yhr045w*Δ and *dap1*Δ is shown as a scatterplot of their NPVs. Every gray point corresponds to one phenotypic screen. Colored crosses highlight phenotypes suggestive of the genes’ shared function. (**B**) Similar to *dap1*Δ, *yhr045w*Δ is sensitive to DNA damaging agents hydroxyurea (HU) and methyl methanesulfonate (MMS). The sensitivity of the *dap1*Δ *yhr045w*Δ double mutant is identical to that of the two single mutants, suggesting that Dap1 and Yhr045w are epistatic to one another. (**C**) Dap1 is one of the five known physical interactors of Yhr045w. (**D**) The sensitivity of *dap1*Δ and *yhr045w*Δ to hydroxyurea and fluconazole is suppressed by the overexpression of *ERG11*. The sensitivity of *dap1*Δ and *yhr045w*Δ to hydroxyurea is suppressed by heme supplementation. (**E**) The similarity of the phenotypic profiles of *ygl117w*Δ and *aro3*Δ is shown as a scatterplot of their NPVs. Every gray point corresponds to one phenotypic screen. Colored crosses highlight phenotypes suggestive of the genes’ shared function. (**F**) The growth of *ygl117w*∆ is severely impaired in tryptophan-limited conditions (SC-Trp) relative to complete media (SC) but is restored in the absence of all three aromatic amino acids (SC-Trp-Tyr-Phe). YPD, yeast extract, peptone, and dextrose; WT, wild type; DMSO, dimethyl sulfoxide.

The first ORF is *YHR045W*, a putative protein of unknown function. Among all mutants in Yeast Phenome, *yhr045w*∆ shows the strongest phenotypic similarity to *dap1∆* (cosine ρ = 0.59 ± 0.07; Fig. 4A) and localizes next to it on the phenotypic similarity map (Fig. 3D). *DAP1* encodes a heme-binding protein that regulates ergosterol biosynthesis and DNA damage response (*23*). One of the phenotypes shared by *dap1*∆ and *yhr045w∆* is sensitivity to hydroxyurea, an inhibitor of DNA synthesis: Both mutants are among the top 15 hits in ~50% of all genome-wide hydroxyurea screens published to date (Fig. 4A and fig. S10A). We experimentally confirmed the sensitivity of *dap1*∆ and *yhr045w*∆ to hydroxyurea (Fig. 4B). We also examined the *dap1∆ yhr045w∆* double mutant and found that the two genes are epistatic to one another, showing nearly identical degree of sensitivity to hydroxyurea alone and in combination (Fig. 4B). Furthermore, Dap1 is one of only five known physical interactors of Yhr045w (Fig. 4C).

Dap1 is thought to regulate ergosterol biosynthesis by stabilizing Erg11, a member of the cytochrome P450 family that catalyzes the demethylation of lanosterol, an essential intermediate in the ergosterol pathway (*24*). The ability of Dap1 to stabilize Erg11 depends on Dap1’s ability to bind heme, an iron-containing complex that serves as a cofactor in numerous cellular reactions, including Erg11’s demethylation activity (*24*). Consistent with their potential joint role in heme binding and Erg11 stabilization, Yeast Phenome data show that *dap1*∆ and *yhr045w*∆ are both sensitive to iron depletion and Erg11 inhibition via chemical compounds such as fluconazole and itraconazole (Fig. 4, A and D). In addition, large-scale genetic interaction screens have shown that *dap1∆* and *yhr045w*∆ are both synthetically lethal with a temperature-sensitive *erg11* mutation (*11*), although the overall genetic interaction profiles of *dap1∆* and *yhr045w*∆ are not correlated (cosine ρ = 0.03 ± 0.11). While the connection between ergosterol biosynthesis and DNA damage is not fully understood, the addition of exogenous heme is able to suppress *dap1∆* and *yhr045w*∆ sensitivity to DNA damage (Fig. 4D), potentially because excess heme availability bypasses a Dap1-Yhr045w requirement for Erg11 stabilization. Consistent with this hypothesis, overexpression of Erg11 also suppresses *dap1*∆ and *yhr045w∆* sensitivity to DNA damage and Erg11 inhibitors (Fig. 4D).

To confirm that the phenotypes observed for *yhr045w∆* are caused by the lack of Yhr045w, we verified its correct genomic sequence in the *Saccharomyces cerevisiae* Genome Variation database (*25*) and complemented its phenotypes with an intact plasmid-borne *YHR045W* (fig. S10B). Together, evidence from Yeast Phenome, genetic interaction and protein-protein interaction data, as well as our validation experiments, suggests that Yhr045w acts in cooperation with Dap1 in regulating DNA damage response and ergosterol biosynthesis. To reflect this joint function, we suggest that *YHR045W* be named *DDE1* for “Dap1-related DNA damage response and ergosterol biosynthesis protein 1.”

### Phenotypic profiles enable dissection of complex pathways

The second ORF we chose to characterize is *YGL117W*, a putative protein of unknown function whose phenotypic profile is highly similar to *aro3*∆ (cosine ρ = 0.62 ± 0.04; Figs. 2C, 3D, and 4E). *ARO3* encodes a 3-deoxy-d-arabino-heptulosonate-7-phosphate (DAHP) synthase, which catalyzes the first step of the chorismate biosynthesis pathway, ultimately producing tryptophan, tyrosine, and phenylalanine (fig. S9A). The phenotypic profile of *ygl117w*∆ is as similar to *trp*∆/*aro*∆ mutants as they are to one another (Fig. 2C and fig. S11A), strongly suggesting that Ygl117w is a new member or regulator of the pathway. Consistent with this hypothesis, and similar to other amino acid biosynthesis genes, *YGL117W* is up-regulated following *GCN4* induction (*26*) and upon amino acid starvation and rapamycin treatment in a *GCN4*-dependent manner (*27*). Furthermore, the promoter of *YGL117W* contains a Gcn4 control response element, which is bound by Gcn4 in vivo (*28*).

We used the *Saccharomyces cerevisiae* Genome Variation database (*25*) to verify that *ygl117w*∆ mutants in YKO are mutated for *YGL117W*. Furthermore, we experimentally confirmed that, similar to *aro3∆* and all *trp*∆ mutants (but not *aro4∆*; see below), the growth of *ygl117w∆* is impaired in tryptophan-limited conditions (Trp^−^; Fig. 4F) and rescued by the expression of a plasmid-borne *YGL117W* (fig. S11B). Despite sharing most other phenotypes with the *trp*∆ mutants, *aro3∆* and *ygl117w∆* are different from the rest of the pathway in that they can grow when all three aromatic amino acids are missing concurrently (Trp^−^ Tyr^−^ Phe^−^; Figs. 2C and 4F). Such difference in growth between Trp^−^ and Trp^−^ Tyr^−^ Phe^−^ media is expected for *aro3*∆, due to Aro3 having functional redundancy with Aro4 (another DAHP synthase) and the feedback inhibition of Aro4 by tyrosine (fig. S9A). However, *aro4*∆ does not mirror this behavior: despite the ability of phenylalanine to inhibit Aro3 activity in vitro, *aro4*∆ exhibits normal growth in Trp^−^ Phe^+^ conditions (Fig. 4F). One possibility is that Ygl117w negatively regulates the feedback inhibition of Aro3 by phenylalanine in vivo and allows *aro4*∆ to maintain DAHP synthesis in Trp^−^ Phe^+^ conditions (fig. S11C). Overall, to reflect the involvement of Ygl117w in the aromatic amino acid biosynthesis pathway, we propose that this gene be named *ARO5*.

### Relationship between phenotypic similarity and intergenic distance

Typically, knockout phenotypes are attributed exclusively to the deleted gene and interpreted as a reflection of its lost function. However, because of the compact nature of the yeast genome [median intergenic distance = 364 base pairs (bp), *n* = 5864], the deletion of one gene can inadvertently disrupt the accessibility and/or regulation of a neighboring nonoverlapping gene. These unintended perturbations, sometimes called neighboring gene effects (NGEs) (*29*), are problematic because they can cause changes in expression and/or localization of nearby proteins and potentially contaminate knockout experiments with incorrect gene-to-phenotype links. For example, in assigning a new function to *YHR045W*, we verified that *yhr045w∆* phenotypes are complemented by *YHR045W* but not *YHR042W*/*NCP1*, a nearby nicotine adenine dinucleotide phosphate (NADP)–cytochrome P450 reductase that is also involved in ergosterol biosynthesis and could be indirectly affected by the deletion of *YHR045W* (fig. S10B). While our data indicate that no such perturbation occurs and *yhr045w∆* phenotypes are due to the loss of *YHR045W*, numerous examples of true NGEs have been reported in the literature (*29*, *30*).

**Fig. 5.**
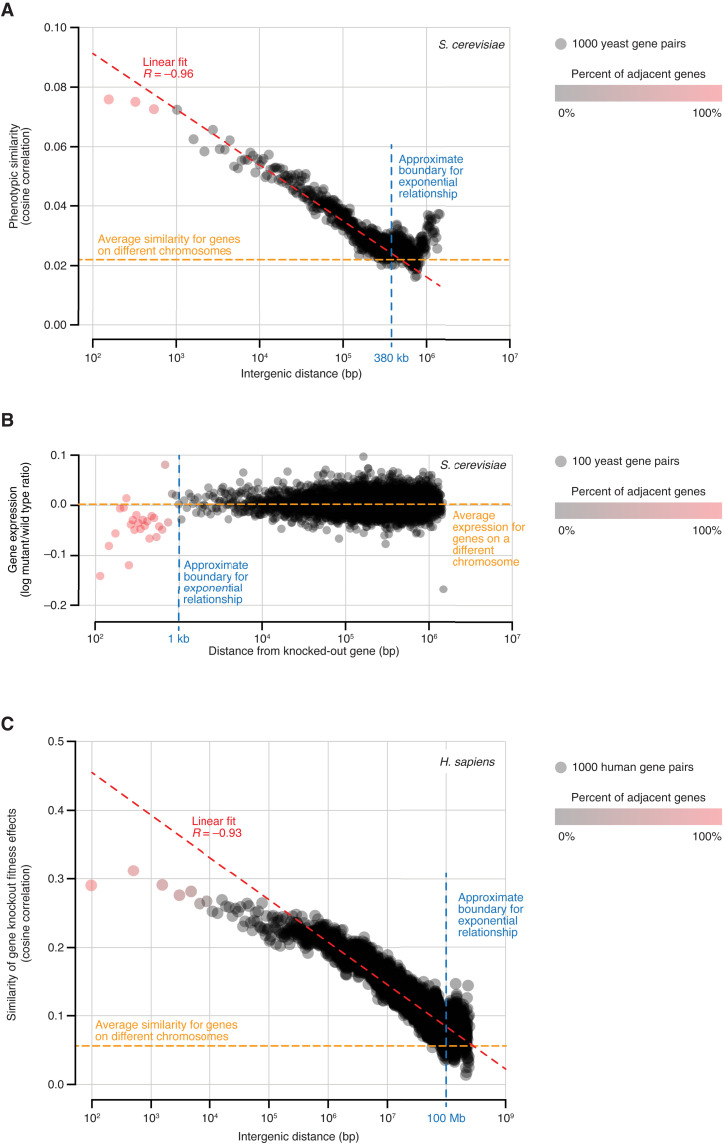
Phenotypic similarity is exponentially related to chromosomal proximity in yeast and human genomes. (**A**) In the yeast genome, the average similarity of phenotypic profiles decays exponentially as a function of intergenic distance. Gene pairs located on the same chromosome were grouped by intergenic distance. In each group, the average intergenic distance and average phenotypic similarity were computed and plotted on the *x* and *y* axes, respectively. (**B**) The effect of the knockout on the expression of nearby genes explains only a small portion of the relationship between intergenic distance and phenotypic similarity. For each knocked-out gene, genes located on the same chromosome were grouped by their distance from the knockout. In each group, the average distance and average change in gene expression in the knockout strain were computed and plotted on the *x* and *y* axes, respectively. (**C**) Similar to yeast, the human genome also displays an exponential relationship between intergenic distance and phenotypic similarity. The analysis was done as described in (A). Phenotypic similarity was estimated by comparing gene effects on fitness across ~1000 cancer cell lines, as measured by genome-wide RNA interference and CRISPR loss-of-function screens.

To systematically measure the extent to which NGEs affect knockout phenotypes, we used Yeast Phenome data to examine the relationship between phenotypic similarity and intergenic distance for ~782,000 gene pairs located on the same chromosome (Materials and Methods). We found that, consistent with potential NGEs, the phenotypic similarity of immediately adjacent genes is significantly higher than that of all other nonoverlapping gene pairs (average cosine ρ = 0.07 versus 0.02, respectively; Kolmogorov-Smirnov test, *P* = 1.5 × 10^−248^; fig. S12). However, to our surprise, excess phenotypic similarity is not limited to adjacent genes: Proximal nonadjacent genes, i.e., those located on the same chromosome but not immediately next to one another, also share significantly more phenotypes than expected (Kolmogorov-Smirnov test, *P* ~ 0.0; fig. S12). A direct comparison of phenotypic similarity and intergenic distance showed a strong exponential relationship such that, for gene pairs located within ~380 kb of one another, closer proximity corresponds to higher phenotypic similarity, and vice versa (Pearson *R* = −0.96, *P* = 2.8 × 10^−283^; Fig. 5A). The same trend was observed independently for each chromosome (fig. S13), as well as for multiple unrelated subsets of the Yeast Phenome dataset (fig. S14).

We asked whether the higher phenotypic similarity between proximal genes can be explained by altered gene expression as would be predicted by NGEs (*29*, *31*). We examined whole-genome transcriptional profiles for ~1500 knockout mutants (*4*) and found that genes immediately adjacent to a knockout are 12 times more likely to change in expression than genes located farther away (0.9% versus 0.08%, respectively; absolute log mutant/wild type ratio |*M*| > 1.7, *P* < 0.05; chi-square test, *P* = 8.3 × 10^−19^). Most adjacent genes (76%) are down-regulated, and, similar to phenotypic similarity, the magnitude of the effect shows an exponential relationship with chromosomal proximity (Fig. 5B). However, the range of this relationship is much shorter than that observed for phenotypic similarity: On average, only genes located within 1 kb from a knockout are affected and 92% of these genes are immediately adjacent to the knockout (Fig. 5B). Such a difference in range between phenotypic similarity and expression effects (380 kb versus 1 kb; Fig. 5, A and B) indicates that, while NGEs may be responsible for increased phenotypic similarity among immediately adjacent gene pairs, the similarity of other genes on the same chromosome is likely driven by other factors.

One possibility is that the phenotypic similarity of proximal genes is due to a batch effect introduced during the construction of knockout mutants. The YKO collection was built by a consortium of 16 laboratories, each responsible for a set of genes in one or more chromosomal regions (*2*). As a result, knockout mutants of proximal genes often originate from the same laboratory and may share additional genetic variation that could enhance their phenotypic similarity. While examples of such laboratory-linked variants have been described (*18*), our analyses indicate that they are relatively rare and underlie a small fraction of gene-gene similarities. For example, proximal genes deleted by each individual laboratory display the same relationship between phenotypic similarity and intergenic distance as all proximal genes (fig. S15). Furthermore, among all genes deleted by the same laboratory, those located on different chromosomes show background levels of phenotypic similarity, whereas those located on the same chromosome are consistently more similar (fig. S16A). The only exceptions to this trend are the 34 knockout mutants on chromosomes II and III, which were generated by laboratory 14 (fig. S16A) and later shown to carry an extra copy of chromosome XI (*18*, *25*). A comprehensive analysis of chromosomal ploidy in ~4400 YKO mutants (*25*) shows that the amplification of chromosome XI in these 34 strains is the only example of chromosome aneuploidy shared by proximal genes (fig. S16B).

In the absence of widespread NGEs and laboratory origin effects, it is possible that the higher phenotypic similarity among proximal genes reflects a closer functional relationship. Several studies in yeast and other organisms have reported evidence for chromosomal colocalization of functionally related genes. In yeast, for example, genes that are coexpressed (*32*, *33*) or co-regulated by the same transcription factor (*34*), as well as genes encoding members of the same protein complex (*35*) or metabolic pathway (*36*), are more likely to be located nearby on the same chromosome than expected by random chance. To assess the extent to which our observations reflect these known trends, we repeated our analysis after excluding ~186,000 gene pairs with existing evidence of functional co-clustering, as well as paralogous genes arisen from an ancient whole-genome duplication event (Materials and Methods). The exponential decay of phenotypic similarity as a function of intergenic distance was unaffected (fig. S17A), indicating that chromosomal location and biological function have a much stronger connection than previously appreciated.

To confirm that our observations are not due to structural changes in the genome caused by gene deletions, we repeated our analysis in native, unperturbed genomes using coexpression across multiple experimental conditions as a measure of functional similarity (*37*). In agreement with previous reports (*32*), we observed that nearby genes are more coexpressed than genes located farther away or on different chromosomes (fig. S17B). In addition, in a manner consistent with phenotypic similarity, average coexpression decayed exponentially as a function of intergenic distance but affected a much shorter range (up to 10.8 kb; fig. S17B).

Last, we asked whether the relationship between intergenic distance and phenotypic similarity is specific to yeast or is conserved in other organisms. The Cancer Dependency Map Project (DepMap) aims to uncover genetic vulnerabilities in human cancers by systematically inactivating genes in a panel of cancer cell lines and measuring the effect of each gene on cell fitness (*38*). Numerous reports have demonstrated that genes sharing similar dependency profiles across cancer cell lines are also likely to share a common function (*39*–*45*). We examined the similarity of dependency profiles for ~8 million human gene pairs located on the same chromosome and observed the same exponential relationship with intergenic distance as in yeast (*R* = −0.93, *P* ~ 0.0; Fig. 5C). This relationship, which extends as far as 100 Mb, is not explained by local chromosomal amplifications that are typical of cancer cell lines and can cause nearby genes to co-vary in copy number and dependency scores (fig. S18) (*46*). The consistency of the effect across Yeast Phenome and DepMap data strongly suggests that, despite differences in genome size, compactness, complexity, and perturbation technologies, yeast and human genomes share one fundamental property: Their genes are not randomly distributed but are positioned relative to one another in a way that reflects their function.

## DISCUSSION

It is commonly assumed that the limiting factor for understanding a biological system is the lack of data or, in some cases, the lack of the right data. Baker’s yeast *S. cerevisiae* is a great example of how inaccurate this assumption might be: Online repositories and the literature are overflowing with data, yet our understanding of the yeast cell as a complete system is still in its infancy. One reason for such a discrepancy between expectation and reality is that data alone are not sufficient to generate knowledge. To be useful, data must generate hypotheses and, to do so, data must be discoverable, understandable, and usable in the context of other types of data (*47*). Yeast Phenome was created to empower integration and reusability of systematic phenotypic screens of the YKO collection and fuel the generation of testable hypotheses. By aggregating, annotating, and harmonizing all available YKO experiments, we have produced an essential dataset for scientists interested in connecting genotypes to phenotypes, predicting gene function, identifying drug targets, understanding the functional principles of genome organization, testing causal inference methods, and answering many other outstanding questions in the systems biology of yeast and other organisms.

Yeast Phenome incorporates and considerably extends all previous efforts to aggregate YKO data (*48*–*51*). In its size, scope, and depth of information, Yeast Phenome rivals many human biobanks that aim to facilitate integrative analyses of human biology by linking genomes, phenomes, and environomes for hundreds of thousands of individuals worldwide (*1*). However, unlike natural populations, where the effect of a variant on gene function must be predicted from sequence and its contribution to a phenotype must be inferred from statistical associations, a knockout screen provides a direct measurement of every gene’s causal effect on a phenotype. While in our current work we focused on complete loss-of-function phenotypes, data libraries similar to Yeast Phenome can be created for phenotypes caused by partial loss-of-function, gain-of-function, dosage-modulating, and point mutations for which genome-wide collections are already available. As part of our aggregation and annotation efforts, we assembled 7011 screens of the yeast heterozygous diploid knockout collection, which capture gene dosage and haploinsufficiency effects on an unprecedented scale. Because of the need to interpret haploinsufficient phenotypes differently from loss-of-function phenotypes, we omitted heterozygous screens from our analyses but are making the dataset available for download and investigation (www.yeastphenome.org; note S3). Additional mutant libraries in alternative genetic backgrounds may also be required to uncover the full spectrum of phenotypes associated with a gene. Despite the availability of numerous examples (*52*, *53*), it is still unclear to what extent the consequences of gene mutations depend on natural variation in the rest of the genome. It is even less clear whether gene-gene phenotypic similarities remain consistent across different backgrounds or whether they change following the reshaping of the underlying functional networks. Answering these questions is important for understanding the rules of genotype-phenotype mapping in yeast and, even more so, in humans.

We have shown that Yeast Phenome helps generating testable hypotheses and improves our understanding of cellular biology. One of the advances enabled by Yeast Phenome is the discovery that more than 1000 chemical compounds, including several drugs approved by the Food and Drug Administration, limit the intracellular abundance of tryptophan (Fig. 2). While *TRP* and *ARO* genes have been previously linked to multidrug resistance in yeast (*7*), the diversity of Yeast Phenome data provides unprecedented insight into a possible mechanism and its relevance to other organisms. It would be tempting to speculate that the compounds eliciting *trp*Δ/*aro*Δ sensitivity bind and inactivate one or both tryptophan permeases (Tat1 and Tat2), therefore inhibiting tryptophan uptake and making the cell dependent on its biosynthesis. However, the chemical structures of *trp*Δ/*aro*Δ compounds are vastly diverse and their known modes-of-action range from rotenone (a mitochondrial complex I inhibitor) and clotrimazole (an ergosterol biosynthesis inhibitor) to ibuprofen (a nonsteroid anti-inflammatory drug) and dehydroepiandrosterone (a human hormone precursor). Such diversity is inconsistent with a direct biochemical interaction with a tryptophan permease or any other protein. A more likely scenario is an indirect effect whereby chemical compounds interfere with tryptophan uptake by changing the structure, composition, or fluidity of the plasma membrane. In support of this hypothesis, ibuprofen has been shown to electrostatically adsorb and then hydrophobically insert into phospholipid bilayers in a dose-dependent manner in vitro (*54*). Physical perturbations that cause *trp*Δ/*aro*Δ sensitivity (low temperature and high hydrostatic pressure; Fig. 2C) are also known to affect membrane fluidity (*55*). Furthermore, most *trp*Δ/*aro*Δ mutants are synthetically lethal with *erg2*–*6*Δ mutants (*11*), which are defective in the production of ergosterol, a primary component of yeast membranes and a regulator of membrane fluidity.

The plasma membrane hosts numerous biomolecules, including sensors, transporters, and enzymes, whose function is sensitive to changes in membrane fluidity. Therefore, it is currently unclear why, relative to all other bioprocesses, tryptophan uptake would be so prominently affected by membrane perturbations. It is possible that the cell is uniquely sensitive to small changes in tryptophan abundance because tryptophan is the largest, rarest, and most energetically expensive of all amino acids (*56*). Furthermore, tryptophan is the only source of de novo nicotinamide adenine dinucleotide (NAD) synthesis and may indirectly regulate many metabolic reactions (*56*). In higher organisms, including humans, tryptophan is the precursor of important neuroactive molecules such as serotonin, melatonin, kynurenine, xanthurenic acid, and quinolinic acid (*56*) and has been implicated in modulating the ability of tumor cells to evade immune surveillance (*57*). Because human cells are unable to synthesize tryptophan and rely completely on dietary intake, the intracellular availability of tryptophan is determined entirely by the regulation of its transport across membranes. The identification of ~1000 chemical compounds that may affect such transport will likely be useful in the investigation of neurological diseases and immuno-oncology.

Another discovery enabled by Yeast Phenome is the exponential relationship between phenotypic similarity and physical proximity among genes located on the same chromosome (Fig. 5). This relationship strongly suggests that genes are not randomly scattered throughout the genome but tend to organize by function. Evidence of co-clustering gene groups has long been available in yeast and other organisms (*32*, *58*, *59*). For example, the major histocompatibility complex (MHC) comprises 20 to 100 related genes located in the same chromosomal region in most vertebrates. Our analyses of Yeast Phenome and human DepMap data indicate that this phenomenon is not limited to isolated blocks of functionally similar genes, such as the MHC. We show that the relationship between gene position and function is much more continuous and long-ranging than previously appreciated (380 kb and 100 Mb in yeast and human genomes, respectively).

One possible explanation for the pervasive genomic colocalization of functionally related genes is the need to efficiently store and access genetic information within the cell nucleus. Given the complexity of DNA packaging and the energetic costs likely associated with selective access to specific DNA regions, it may not be unexpected that the genes often accessed together are positioned nearby. Another possible explanation is that physical proximity among functionally related genes has evolutionary advantages for maintaining favorable combinations of alleles. It has been proposed that, when two alleles share a genetic interaction (i.e., their joint effect on fitness is greater than the sum of their individual effects), natural selection should act to preserve the successful haplotype and suppress recombination between the two loci (*59*, *60*). Given that functionally related genes are strongly enriched for genetic interactions (*9*, *11*), it is possible that their relative genomic positions are under selective pressure to reduce recombination rate and enhance genetic linkage by minimizing physical distance.

A recurrent theme that emerged from our analyses is the importance of examining phenotypic profiles in addition to individual gene-phenotype pairings. A phenotypic profile, intended either as a set of phenotypes associated with a gene or as a set of genes associated with a phenotype, is a powerful tool for investigating a biological system because it is quantitative, comprehensive, and robust to noise. This global perspective is often missed by studies that focus on characterizing only the strongest hits from a loss-of-function screen or, in a largely similar manner, only the most statistically significant variants from a genome-wide association study. It is becoming increasingly clear that great value can be derived from examining all genetic variations linked to a trait and all traits linked to a genetic variant, regardless of their significance against an arbitrary threshold.

## MATERIALS AND METHODS

### Data sources

A detailed description of strategies used to collect, annotate, and normalize Yeast Phenome data is provided at https://yeastphenome.org/about/project/. A list of additional datasets used for analysis is provided in note S4.

### Calculating profile similarity

To compute robust, outlier-insensitive, gene-gene similarities and corresponding confidence estimates in a computationally efficient and parallelizable manner, we adopted the following bootstrap strategy. Given a data matrix (e.g., the Yeast Phenome dataset), where rows correspond to genes and columns correspond to gene features (e.g., phenotypic screens), we created 100 submatrices by selecting 1500 columns (~10%) using random sampling with replacement. For each of the submatrices, we computed gene-gene correlations as defined by a chosen similarity metric (e.g., cosine, Pearson, or Spearman correlations) using a parallelized implementation by deepgraph (*61*). For each gene pair, we combined the 100 sampled correlations and computed the mean and SD.

### Analysis of reproducibility among near-replicate screens

Two screens were considered near-replicates if they shared the same phenotype, the same condition, and the same media. In this analysis, all types of growth measurements (e.g., colony size, culture turbidity, and relative abundance of pooled culture) were considered as one phenotype. Media containing common buffers (e.g., HEPES) or solvents (e.g., dimethyl sulfoxide) were also considered to be equivalent to the same media without the additions.

### Precision-recall analysis of profile similarities

Data for the functional groups (GO biological process terms, protein complexes, and biochemical pathways), genetic interactions, protein-protein interactions, and gene expression were obtained as described above (see the “Data sources” section). Profile similarities (ρ) were computed as described in the notes for each data type. Gene pairs were sorted (highest to lowest) by the similarity of their profiles. Recall was defined as the number of functionally related gene pairs with ρ > α (for decreasing values of α). Precision was calculated as the fraction of functionally related gene pairs among all gene pairs with ρ > α. In the global analysis, a gene pair was considered functionally related if both genes were co-annotated to the same GO biological process term, protein complex, or biochemical pathway. In the stratified analysis, a gene pair was considered functionally related if both genes were co-annotated to a functional group from a specific set (e.g., only GO biological process terms). AUPR was calculated as the ratio between the area under the true precision-recall curve and the area under an ideal precision-recall curve, which would occur if all functionally related gene pairs were ranked higher than all other gene pairs.

### Principal components analysis

A Yeast Phenome data matrix (4554 genes × 8372 screens) was prepared by excluding gene expression data from Kemmeren *et al.* (*4*) because only ~1500 knockout mutants were tested. The principal components (PCs) were computed using eigendecomposition of the covariance matrix, implemented in NumPy. Knockout mutants projected on the first PC were examined using GO slim annotations. Specifically, we identified GO terms such that at least 50% of their members were present among the top 20% genes within the (i) highest or (ii) lowest coordinates in PC1. These GO terms included the following: (i) Golgi vesicle transport, endocytosis, endosomal transport, exocytosis, and vesicle organization and (ii) cytoplasmic translation, mitochondrial translation, ribosomal RNA processing, ribosomal large and small subunit biogenesis, ribosomal subunit export from nucleus, ribosome assembly, and tRNA aminoacylation for protein translation. Knockout projections on the second PC were compared to mutant growth rate, which was estimated by averaging gene-specific NPVs across 10 screens of growth in standard laboratory conditions (screen IDs 26, 540, 758, 4776, 5257, 5395, 16183, 16187, 16487, and 16490).

### Phenotype rate analysis

Phenotype rate for a knockout mutant *i* was defined as *P_i_* = *N*_s_/*N*, where *N* is the total number of screens in which the knockout *i* was tested and *N*_s_ is the number of screens in which knockout *i* displayed a strong phenotype, i.e., |NPV| > 3. To avoid biases, gene expression data from Kemmeren *et al.* (*4*) were excluded from this analysis because only ~1500 knockout mutants were tested.

### Constructing the phenotypic similarity map

The Yeast Phenome data matrix was restricted to 1586 genes with at least 1% phenotype rate and 8260 phenotypic screens with more than 1500 tested mutants [i.e., we excluded the gene expression data from Kemmeren *et al.* (*4*)]. All genes were projected onto a 2D space using the Python implementation of Uniform Manifold Approximation and Projection (UMAP) (*62*) with the following parameters: *n_neighbors* = 10, *min_dist* = 0.75, n_*components* = 2, and *metric* = ‘cosine’.

The UMAP was annotated using GO Slim biological process terms (see the “Data sources” section) and a modified version of SAFE (*5*). Briefly, for each gene, we define a local neighborhood as the set of genes located with a Euclidean distance of *d* from it. In this case, *d* was defined as 7% of the map diameter, i.e., the maximum distance between two genes on the map. At this *d* threshold, a typical neighborhood included 41.03 ± 14.28 genes (mean ± SD). Each neighborhood is tested for enrichment for all GO terms using a standard Fisher’s exact test. The GO term with the lowest enrichment *P* value is assigned to the gene at the center of the neighborhood.

SAFE was also applied for annotating the UMAP with the results of quantitative phenotypic screens. Each phenotypic screen is associated with a set of NPVs. The NPVs for all genes in a neighborhood were summed to produce a neighborhood phenotypic value Σ_NPV_. The NPVs were then randomized 1000 times, and a distribution of random cumulative phenotypic values was produced for each neighborhood. An empirical enrichment *P* value was calculated by comparing the observed phenotypic values (Σ_NPV_) to the random ones. The *P* value can be interpreted as the probability of observing a neighborhood phenotypic value as high or higher than Σ_NPV_ by random chance (and the opposite for lower values).

### Analysis of phenotypic similarity versus intergenic distance

Chromosomal coordinates for all genes in the yeast genome were obtained as described in the “Data sources” section. Intergenic distance was calculated as the difference between the leftmost coordinate of the upstream gene and the rightmost coordinate of the downstream gene. The relationship between intergenic distance and phenotypic similarity was examined by sorting all gene pairs by their distance, splitting the genes into groups of 1000 pairs each and computing the average distance and phenotypic similarity within each group.

The upper distance boundary for excess phenotypic similarity (i.e., the inflection point in the relationship between phenotypic similarity and intergenic distance) was estimated as follows: (i) Compute a Pearson correlation coefficient between the intergenic distance (on a log scale) and phenotypic similarity for all gene pairs located within a distance *d*, (ii) repeat the calculation for a range of *d* values, and (iii) choose the value of *d* that corresponds to a local optimum of Pearson correlation (i.e., the negative peak with a range of similar *d* values). The same approach was used for analyzing gene expression and human gene knockout data.

To examine the impact of gene pairs with existing evidence of functional co-clustering, we excluded from the analysis all gene pairs that fit any of the following criteria (see the “Data sources” section): (i) Gene pairs co-annotated to the same protein complex, (ii) gene pairs co-annotated to the same biochemical pathway, (iii) gene pairs co-annotated to the same moderately specific GO biological process term, (iv) gene pairs in the 75th percentile of coexpression values (cosine ρ > 0.15), (v) genes co-regulated by the same transcription factor, and (vi) duplicated gene pairs. To examine the potential effect of gene copy number amplification in human cancer cell lines, we identified 5423 genes that present a copy number greater than 4 in at least three of the cancer cell lines used in our analysis (see the “Data sources” section). All correlations involving any of these genes were removed from the analysis.

### Strain construction

All single-deletion mutants used for validation, as well as an isogenic wild-type control, were taken from the Prototrophic Deletion Collection (table S5) (*63*). The double deletion for *dap1*∆ *yhr045w*∆ (ABY004) was constructed by transforming *dap1*∆ (ABY002) with a NatMX cassette polymerase chain reaction (PCR)–amplified from pRS40_Nat/pFvL099 (*64*). The amplification primers (Nat_F and Nat_R; table S6) contained 40 bp of the flanking region (upstream and downstream) of *YHR045W*. The double deletion was confirmed by PCR and selection on clonNat and G418. Strains overexpressing *ERG11* were constructed by transforming wild type (ABY001), *dap1*∆ (ABY002), and *yhr045w*∆ (ABY003) with either an *ERG11* multicopy 2-μm plasmid or empty vector from the MoBY-ORF 2.0 collection (*65*). All transformations were confirmed by leucine selection.

Plasmids used for *yhr045w*∆ complementation assays were constructed using a pRS412-NatNT2 plasmid backbone. The natNT2 fragment from pFA6a-natNT2 was PCR-amplified with primers incorporating both 5′ and 3′ BglII restriction sites (natNT2_F and natNT2_R; table S6). This fragment was inserted into pRS412 cut with BglII to remove the adenine (ADE) selection site. The *NCP1* and *DAP1* plasmids for complementation were constructed by amplifying the entire ORF by PCR using oligonucleotide primers (NCP1_HindIII, NCP1_XbaI, DAP1_HindIII, and DAP1_XbaI; table S6) that incorporate 5′ HindIII and 3′ XbaI restriction enzyme sites to allow cloning into the pRS412-NatNT2 vector. The *YHR045W* ORF was synthesized from GenScript and inserted into a pUC57-Mini plasmid. The pRS412-NatNT2 and pUC57-Mini-*YHR045W* plasmids were amplified, digested with NotI and EcoRV restriction sites, and ligated before direct transformation into *Escherichia coli*. All plasmids were confirmed by Sanger sequencing before transformation into the deletion strains.

The plasmid used for *ygl117w*∆ complementation was taken from the MoBY-ORF 2.0 collection (*65*). Transformation was confirmed by leucine selection.

### Spot assays

Single colonies were grown overnight in 5 ml of yeast extract, peptone, and dextrose (YPD) culture until saturation. They were diluted to an optical density of 1.0 in the morning and washed three times with water. The cells were then resuspended with 1 ml of YPD. A series of 10-fold serial dilutions were prepared in a 96-well round-bottom plate and spotted using a sterilized 48- or 96-pin pronger onto the appropriate growth medium. All experiments were repeated at least twice (biological replicates).

### Media

#### YGL117W validation

Cells were spotted on either synthetic complete (SC), SC-Trp, or SC-Trp-Tyr-Phe agar plates. All media contained yeast nitrogen base with ammonium sulfate (5 g/liter) supplemented with 2% glucose. SC and SC-Trp media were supplemented with SC mix (2 g/liter; Sunrise, catalog no. 1300) and SC-Trp (1.98 g/liter; Sunrise, catalog no. 1305), respectively. For SC-Trp-Tyr-Phe, amino acids were prepared and added back individually according to the concentrations listed in the description of synthetic complete media (Sunrise, catalog no. 1300), but excluding tryptophan, tyrosine, and phenylalanine.

#### YHR045W validation

Yeast extract and peptone (YP) agar (1.1×) was microwaved for approximately 3 to 5 min until fully boiled. The solution was then cooled to 50°C in a water bath, and 20% glucose stock solution was added to a final concentration of 2%. Forty milliliters of media was poured into each empty OMNI plate, and chemical compounds (from Sigma-Aldrich) were added at the following concentrations: fluconazole (35 μM), heme (20 μM), hydroxyurea (80 mM), itraconazole (35 μM), and methyl methanesulfonate (0.020%).

## Supplementary Materials

### This PDF file includes:

Notes S1 to S4 Figs. S1 to S18 Tables S1 to S6 References
