# Supplementary material for: Global analysis of the yeast knockout phenome
Source: Sci Adv. 2023 May 26;9(21):eadg5702. doi: 10.1126/sciadv.adg5702 (PMC11326039; doi:10.1126/sciadv.adg5702)
Supplement: Supplementary file 1 — Notes S1 to S4 Figs. S1 to S18 Tables S1 to S6 References [file sciadv.adg5702_sm.pdf]

Science Advances
AAAS

Supplementary Materials for

Global analysis of the yeast knockout phenome

Gina Turco *et al.*

Corresponding author: Anastasia Baryshnikova, abaryshnikova@calicolabs.com

Sci. Adv. **9**, eadg5702 (2023)
DOI: 10.1126/sciadv.adg5702

This PDF file includes:

Notes S1 to S4
Figs. S1 to S18
Tables S1 to S6
References

Note S1 – Reproducibility of YKO screens

By virtue of its size and robust annotations, Yeast Phenome enables an assessment of knock-out screen reproducibility. An example of an experiment repeated multiple times is growth on glycerol, a non-fermentable carbon source that can only be metabolized via respiration. Inability of knock-out mutants to respire, revealed by their failure to proliferate on rich media containing glycerol as the sole carbon source, has been systematically tested 8 times in 6 different laboratories (table S1). By comparing the results of all 8 screens, we found that each screen reported a similar number of slow growing mutants (NPV < –3) that were deemed respiration deficient (140 ± 42 mutants, mean ± std. dev., n = 8). Importantly, most mutants (57–82%) identified in any one screen were reproduced in at least 5 of the 8 independent replicates, while 0–24% of mutants remained unique to single datasets (fig. S2A).

One possible explanation for genes required for respiration in some but not all screens is that differences in experimental design (e.g., glycerol dosage, type of growth assay, mutant ploidy or mating type) create conditional essentialities. If that is the case, we expect genes unique to any single experiment to share common biological functions as often as genes reproduced in multiple experiments. To test this hypothesis, we used proximity on the genetic interaction similarity (GIS) network as an unbiased measure of shared function. The GIS network connects genes if their mutations have similar effects on the fitness of other mutants (*11*). Without any prior knowledge of gene function, the GIS network places genes relative to one another based on the extent of their functional similarity, producing an unsupervised view of cellular organization spanning multiple levels of resolution, from molecular pathways to organelles (*5*, *11*). We performed Spatial Analysis of Functional Enrichment (SAFE) (*5*) to identify regions of the GIS network that are overrepresented for respiration-deficient mutants from each screen (fig. S2B). We found that the enrichment profiles of all screens were nearly identical to one another (cosine correlation between neighborhood enrichment scores $\rho = 0.994 \pm 0.003$, mean ± std. dev., n_{pairs} = 28) and consistent with respiration, oxidative phosphorylation, and other mitochondrial and metabolic functions (fig. S2B). The enrichment was driven primarily by mutants identified in multiple screens, whereas mutants unique to single experiments scattered randomly throughout the network (fig. S2C). This observation does not support the hypothesis that isolated findings share common biological functions and suggests, instead, that they are likely false positives. Furthermore, it suggests that, whenever replicate screens are not available, SAFE enrichment profiles could inform our level of confidence in single observations.

Note S2 – Analysis of secondary mutations

Knock-out collections are built on the principle that genetic loci can be systematically altered, one at a time, while keeping the rest of the genome constant. However, the genomes of knock-out mutants are not expected to remain constant over generations. Depending on time and selective pressure, knock-out mutants may acquire secondary mutations that partially compensate for gene loss and alleviate any corresponding fitness defects. Consistent with this expectation, studies have reported heritable phenotypic heterogeneity within isogenic knock-out populations (*83*), mapped secondary site suppressors using systematic genetic crosses (*84*) and identified a wide range of genomic alterations through whole genome sequencing (*18*, *25*). While knowing that adaptation is a general property of living systems that can be minimized but not eliminated completely, we sought to understand to what extent acquired secondary mutations may affect the interpretation of phenotypes derived from the yeast knock-out collection.

We compared phenotypes across two independently constructed versions of the haploid collection (Mat-a and Mat-α), as well as the homozygous diploid collection produced by mating them. Copies of the collections were housed separately across many laboratories and exposed to vastly different experimental conditions, giving each strain an opportunity to evolve independently from its siblings. Despite the opportunity to diverge, estimates of phenotype rate, i.e. the frequency of strong phenotypes ($|NPV| > 3$) displayed by each gene (see also Fig. 2 and related section in the main text), were correlated across collections (e.g., cosine $\rho = 0.66–0.72$; fig. S4A), suggesting that secondary mutations are either rare, reoccur frequently in strains lacking the same gene or have relatively little impact on most phenotypes.

To examine the impact on phenotypes more directly, we asked how often secondary mutations mask existing phenotypes or produce new ones, thus lowering the degree to which a phenotypic profile reflects the function of the deleted gene. Using data from previous investigations (*83*, *84*), we compiled a list of 207 knock-out mutants that show ($n = 103$) or do not show ($n = 104$) evidence of secondary mutations (Materials & Methods). We presented random subsets of this list to two independent examiners and asked them to evaluate the phenotypes of each gene with respect to the gene's known biological function (Materials & Methods). The evaluations provided by each examiner, as well as their consensus, showed no statistical association between evidence of secondary mutations and phenotype-function inconsistency (p-value = 0.98, one-tailed Fisher's exact test; fig. S4B). Indeed, in contrast to expectation, the phenotypes of knock-out mutants carrying secondary mutations were more, not less, likely to agree with the functions of the deleted genes (70% among strains with secondary mutations vs 58% in the control group; fig. S4B). Given these data, we estimate with 95% confidence that secondary mutations increase the relative risk of phenotype-function inconsistency by no more than 3% (relative risk RR = 0.711, 95% CI [0.491, 1.030]; table S2).

The relatively low impact of secondary mutations on the functional interpretation of knock-out phenotypes may be explained by close functional proximity (and therefore high phenotypic similarity) between the two affected genes. Indeed, in cases where secondary mutations have been identified, they often occurred in genes that act in the same biological pathway, protein complex or regulatory response as the deleted gene (*83*, *84*). These observations suggest that secondary mutations are likely to modulate, but not obscure, the

phenotypes of the original deletion. Consistent with this hypothesis, knock-out mutants with and without secondary site suppressors show highly similar genetic interaction profiles (*84*). We therefore conclude that secondary mutations, arising spontaneously during routine laboratory manipulations, should not impede the use and interpretation of phenotypic profiles derived from the yeast knock-out collection.

Note S3 – Phenotypic screens of the heterozygous diploid YKO

The data provided at www.yeastphenome.org and used for analysis in this study include only phenotypic screens of the two haploid YKO collections (Mat-a and Mat-α) and the homozygous diploid YKO collection. However, we also assembled and annotated data derived from phenotypic screens of the heterozygous diploid collection. These data were processed and transformed in the same manner as the haploid/homozygous phenotypic screens (Materials & Methods). The unprocessed input data and processing code for each publication reporting heterozygous screens are provided in the yp-data Github repository (https://github.com/yeastphenome/yp-data) and its archived version (https://doi.org/10.5281/zenodo.7714347). In addition, we're providing the following 3 files in the "Bundle downloads" section of the Yeastphenome.org website (https://yeastphenome.org/downloads/):

1. yp_datasets_het_20221018.tar.gz (tab-delimited) – A list of heterozygous diploid screens with relevant metadata (explained in the README.txt file).
2. yp_matrix_het_20221018.tar.gz (tab-delimited) – A gene x screen matrix for cleaned but unnormalized phenotypic values.
3. yp_matrix_het_z_20221018.tar.gz (tab-delimited) – A gene x screen matrix of normalized phenotypic values.

Note S4 – List of datasets used for analysis

Yeast Phenome
Versions: 2022-02-08 and 2022-10-25
URL: https://yeastphenome.org/downloads/
Notes:

1. Similarity of phenotypic profiles was measured for each pair of genes using a bootstrap strategy as described below ("Calculating profile similarity"). The similarity metric was cosine correlation. Gene expression data from Kemmeren et al., 2014 (*4*) were excluded from similarity analyses because only ~1,500 knock-out mutants were tested.
2. When comparing phenotypic profiles to genetic interaction, protein-protein interaction and gene expression profiles, genes encoding ribosome components were excluded.

Genetic interactions
Publication: Costanzo et al., 2016 (*11*)
Notes:

1. Similarity of genetic interaction profiles was measured for each pair of query strains using a bootstrap strategy as described below ("Calculating profile similarity"). The similarity metric was cosine correlation. Dubious ORFs, essential genes and genes encoding ribosome components were excluded.
2. Genetic interaction degree was calculated as the number of genetic interactions per query strain that satisfy the intermediate stringency cutoff: $|\varepsilon| > 0.08$ and p-value < 0.05 (where ε is the genetic interaction score).

Protein-protein interactions
Database: BioGRID (*85*)
URL: https://downloads.thebiogrid.org/File/BioGRID/Release-Archive/BIOGRID-4.3.195/BIOGRID-ORGANISM-4.3.195.tab3.zip
Accessed on: 2021-03-31
Notes:

1. Similarity of protein-protein interaction profiles was measured a bootstrap strategy as described below ("Calculating profile similarity"). The similarity metric was Jaccard index. Dubious ORFs, essential proteins, ribosome components and proteins with fewer than 4 interactions were excluded.

Gene expression
Database: SPELL (*37*)
URL: http://sgd-archive.yeastgenome.org/expression/microarray/
Accessed on: 2017-09-24
Notes:

1. Similarity of gene expression profiles was measured using a bootstrap strategy as described below ("Calculating profile similarity"). The similarity metric was cosine correlation. Dubious ORFs, essential genes and genes encoding ribosome components were excluded.

Transcription factor/target gene mapping

Publication: Balaji et al., 2006 (*86*)

Protein complexes
Database: EMBL-EBI Complex Portal
URL: http://ftp.ebi.ac.uk/pub/databases/intact/complex/current/complextab/559292.tsv
Accessed on: 2022-11-02

Biochemical pathways
Database: Yeast Biochemical Pathway Database (YeastCyc) via SGD (*20*)
URL: http://sgd-archive.yeastgenome.org/curation/literature/biochemical_pathways.tab
Accessed on: 2022-03-02

Gene Ontology (GO)
Database: Gene Ontology Consortium
URL: http://geneontology.org/
Accessed on: 2017-12-06
Notes:

1. In the precision-recall analysis of phenotypic profiles, as well the analysis of chromosomal co-clustering, GO was restricted to a list of 295 biological process terms that were previously identified by expert biologists as moderately specific (*87*).

Multifunctional genes
Database: SGD (*20*)
URL: http://sgd-archive.yeastgenome.org/curation/literature/go_slim_mapping.tab
Accessed on: 2021-05-04
Notes:

1. A gene was defined as "multifunctional" if it was annotated to 4 or more different GO Slim terms.

Duplicated gene pairs
Publication: Byrne et al., 2005 (*88*)

Conserved genes
Database: The Alliance of Genome Resources (*89*)
URL: https://www.alliancegenome.org/
Accessed on: 2022-06-28
Notes:

1. A *S. cerevisiae* gene was defined as conserved if it had an ortholog (i.e., best reciprocal match) in *D. rerio*, *M. musculus*, *R. norvegicus*, *C. elegans*, *D. melanogaster* or *H. sapiens*.

Uncharacterized ORFs
Database: SGD (*20*)
URL:
https://yeastmine.yeastgenome.org/yeastmine/bagDetails.do?scope=all&bagName=Uncharacterized_ORFs

Accessed on: 2022-09-03

Gene coordinates (S. cerevisiae)
Database: SGD (*20*)
URL: http://sgd-archive.yeastgenome.org/curation/chromosomal_feature/SGD_features.tab
Accessed on: 2017-04-03

Human gene knock-out data
Database: The Cancer Dependency Map Project (DepMap) (*38*)
URL: https://depmap.org/portal/download/all/ [CRISPR_gene_effect.csv]
Version: 22Q2
Accessed on: 2022-10-10
Notes:

1. The similarity of gene effects was calculated using cosine correlation implemented in deepgraph (*61*).

Gene copy number of human cancer cell lines
Database: The Cancer Dependency Map Project (DepMap) (*38*)
URL: https://depmap.org/portal/download/all/ [CCLE_gene_cn.csv]
Version: 22Q2
Accessed on: 2023-01-28

Gene coordinates (H. sapiens)
Database: Gene (NCBI)
URL: https://www.ncbi.nlm.nih.gov/gene
Accessed on: 2022-10-11

Laboratory origin of YKO strains
Publication: Hoepfner et al., 2014 (*18*)

Table S1 – List of Yeast Phenome screens for respiratory metabolism

Screen ID	Paper	Collection	Growth assay	Medium	Data type	Notes
470	Dimmer et al., 2002 (*76*)	Hom	Undefined	YPG 2%	Discrete	
4837	Steinmetz et al., 2002 (*77*)	Hom	Pooled culture	YPG 3%	Quantitative	
5000	Dudley et al., 2005 (*78*)	Hom	Spot assay	YPG 3%	Discrete	
417	Luban et al., 2005 (*79*)	Hap a	Undefined	YPG 3%	Discrete	
21955	Kuepfer et al., 2005 (*69*)	Hap a	Undefined	MM (Verduyn mix) + His + Leu + Met + Ura, Glycerol (2%)	Discrete	Excluded from analysis (SC medium)
1050, 1051	Hillenmeyer et al., 2008 (*7*)	Hom	Pooled culture	YPG	Quantitative	Excluded from analysis (unknown YPG dose)
158	Merz et al., 2009 (*80*)	Hap α	Spot assay	YPG 3%	Discrete	
16489	Qian et al., 2012 (*81*)	Hom	Pooled culture	YPG 5%	Quantitative	
16369, 16388	Galardini et al., 2019 (*52*)	Hap a	Colony size	SC + G 2%	Quantitative	Excluded from analysis (SC medium)
22018	Acton et al., 2017 (*66*)	Hom	Pooled culture	YPG 3%	Quantitative	
21874	Stenger et al., 2020 (*82*)	Hap a	Colony size	YPG 3%	Discrete	

Only experiments that employed YP-based media and glycerol as the sole carbon source were included in this analysis. Yeast Phenome contains additional data for growth on synthetic media (partial and complete) and media supplemented with other carbon sources (e.g., glucose, ethanol).

Table S2 – Analysis of phenotype-function consistency for knock-out mutants carrying secondary mutations

		Phenotype-function consistency			
		**Yes**	**No**	**Total**	**Risk**
**Secondary mutations**	**Yes**	A = 72<br>$A_0 = 66$	B = 31<br>$B_0 = 37$	103	ARM = b / (a+b) = 0.3
	**No**	C = 60<br>$C_0 = 66$	D = 44<br>$D_0 = 38$	104	ARW = d / (c+d) = 0.42
	**Total**	132	75	207	

The relative risk (RR) of a secondary mutation affecting the phenotypes is given by RR = ARM / ARW = 0.3 / 0.42 = 0.711 (values below 1 indicate that secondary mutations help, do not harm, phenotypic profiles).
To estimate the confidence intervals around this relative risk, we can use the Taylor series approximate variance (*90*). The two-sided 95% confidence limits are given by:

$$CI = e^{lnRR \pm 1.96\sqrt{\frac{1-ARM}{b}+\frac{1-ARW}{d}}}$$

So, in this case, RR = 0.711, CI 95%: [0.491, 1.030]. That means that, with 95% confidence, the relative risk of a secondary mutation to negatively impact the phenotypic profile of a knock-out mutant is at most 3%.

Table S3 – List of screens with the highest number of chemogenomic associations

Screen ID	Phenotype / Condition	Paper	Number of chemogenomic associations
699	unfolded protein response (UPR) (UPRE-GFP) / YPD	Jonikas et al., 2009 (*19*)	692
16429	vacuole class G (Vph1) / SD [low-fluor] + Met + Leu + Ura + NAT + G418	Mattiazzi Usaj et al., 2020 (*91*)	334
16550	protein stability (tFT-Tom5) / SC - Leu + Ade	Dederer et al., 2019 (*92*)	189
16324	mtDNA copy number (mtDNA/whole genome)	Puddu et al., 2019 (*25*)	175
16428	vacuole fragmented (Vph1) / SD [low-fluor] + Met + Leu + Ura + NAT + G418	Mattiazzi Usaj et al., 2020 (*91*)	122
21877	expression of mtDNA (ARG8m) / SC - Arg	Stenger et al., 2020 (*82*)	118
4802	abundance of potassium / SC + element supplements	Yu et al., 2011 (*93*)	111
5356	chronological lifespan / glucose [0.5%], time [9 d], SC, Hopkins (-C)	Matecic et al., 2010 (*94*)	103
4949	abundance of glycogen (iodine staining) / SC	Wilson et al., 2002 (*95*)	103
1190	abundance of calcium / hypotonic shock, DCD - Leu	Loukin et al., 2007(*96*)	93

Table S4 – List of uncharacterized ORFs with at least 1% pleiotropy and strong profile similarity to verified ORFs

	Top correlated gene				
**Uncharacterized ORF**	**ORF**	**Gene name**	**Correlation (mean)**	**Correlation (std. dev.)**	**Phenotype rate (%)**
YLR261C	*YLR262C*	*YPT6*	0.689	0.05	0.13
YBR062C	*YGL131C*	*SNT2*	0.667	0.034	0.01
YGL117W	*YDR354W*	*TRP4*	0.66	0.031	0.04
YHR045W	*YPL170W*	*DAP1*	0.582	0.068	0.03
YDR114C	*YER050C*	*RSM18*	0.535	0.038	0.03
YIL077C	*YIL041W*	*GVP36*	0.515	0.099	0.02
YIL029C	*YOR043W*	*WHI2*	0.489	0.065	0.02
YKR073C	*YKR072C*	*SIS2*	0.476	0.037	0.02
YIL014C-A	*YIL060W*	*YIL060W*	0.471	0.037	0.02
YFL034W	*YHL019C*	*APM2*	0.458	0.081	0.02
YNL184C	*YNL252C*	*MRPL17*	0.445	0.046	0.03
YHL029C	*YNL056W*	*OCA2*	0.441	0.05	0.01
YCR087C-A	*YCR045C*	*RRT12*	0.439	0.04	0.02
YJL193W	*YJL155C*	*FBP26*	0.437	0.073	0.02
YLL030C	*YIL121W*	*QDR2*	0.418	0.065	0.01
YPL056C	*YPL057C*	*SUR1*	0.417	0.125	0.01
YCR050C	*YCR045C*	*RRT12*	0.417	0.047	0.03
YGL088W	*YDR450W*	*RPS18A*	0.412	0.033	0.02
YLR331C	*YLR332W*	*MID2*	0.405	0.069	0.02
YOR183W	*YPR106W*	*ISR1*	0.396	0.035	0.03
YCL001W-A	*YCR026C*	*NPP1*	0.395	0.045	0.03
YCR007C	*YDL010W*	*GRX6*	0.391	0.059	0.01
YNL140C	*YHR167W*	*THP2*	0.387	0.057	0.03
YPR089W	*YDL226C*	*GCS1*	0.364	0.06	0.01
YBR027C	*YDL066W*	*IDP1*	0.362	0.049	0.01
YCR085W	*YBR295W*	*PCA1*	0.356	0.045	0.01
YEL033W	*YIR026C*	*YVH1*	0.352	0.035	0.03
YKL121W	*YBR208C*	*DUR1,2*	0.347	0.051	0.01
YER077C	*YNL177C*	*MRPL22*	0.346	0.039	0.04
YGL007W	*YMR216C*	*SKY1*	0.341	0.041	0.05
YBR284W	*YCR045C*	*RRT12*	0.336	0.047	0.02
YEL028W	*YBR149W*	*ARA1*	0.33	0.064	0.01
YCR025C	*YCR045C*	*RRT12*	0.329	0.042	0.01

YLR426W	*YDL066W*	*IDP1*	0.327	0.035	0.04
YER084W	*YDL100C*	*GET3*	0.326	0.047	0.01
YMR262W	*YDR458C*	*HEH2*	0.322	0.047	0.01
YML037C	*YPR029C*	*APL4*	0.319	0.044	0.01
YCR051W	*YBR278W*	*DPB3*	0.314	0.037	0.01
YLR358C	*YJL080C*	*SCP160*	0.31	0.046	0.06
YML122C	*YLR372W*	*ELO3*	0.304	0.041	0.12
YLR407W	*YJR083C*	*ACF4*	0.299	0.062	0.01
YDR525W	*YOR085W*	*OST3*	0.296	0.044	0.02
YHL044W	*YJL192C*	*SOP4*	0.29	0.04	0.01
YIL067C	*YCR009C*	*RVS161*	0.238	0.045	0.02
YHL005C	*YMR256C*	*COX7*	0.236	0.042	0.01
YLR125W	*YDL184C*	*RPL41A*	0.176	0.032	0.01

Table S5 – List of yeast strains used for validation experiments

Strain ID	Genotype
Yeast knock-out collection (YKO)	MATa *orf*Δ::KanMX *his3*Δ1 *leu2*Δ0 *met15*Δ0 *ura3*Δ0
Prototrophic deletion collection (PDC)	MATa *orf*Δ::KanMX *can1*Δ::*STE2pr*-Sp_*his5* *his3*Δ1 *lyp1*Δ0
ABY001	PDC hoΔ::KanMX
ABY002	PDC *dap1*Δ::KanMX
ABY003	PDC *yhr045w*Δ::KanMX
ABY004	PDC *ygl117w*Δ::KanMX
ABY005	PDC *dap1*Δ::KanMX *yhr045w*Δ::NatMX
ABY006	ABY001 [MoBY-2μ-*LEU2*-*ERG11*]
ABY007	ABY002 [MoBY-2μ-*LEU2*-*ERG11*]
ABY008	ABY003 [MoBY-2μ-*LEU2*-*ERG11*]
ABY009	ABY002 [pRS412-NatNT2-*DAP1*]
ABY010	ABY003 [pRS412-NatNT2-*YHR045W*]
ABY011	ABY003 [pRS412-NatNT2-*NCP1*]
ABY012	ABY004 [MoBY-2μ-*LEU2*-*YGL117W*]

Table S6 – List of primers

Primer	Sequence
Nat_F	ACTATTTGGCCAAGACGGTTGCTTATACATCTATAATCAAAGATTGTACT GAGAGTGCAC
Nat_R	GATAATAATAAATAATAAATAATAAAGCGATAGATAATGCCTGTGCGGT ATTTCACACCG
natNT2_F	AAAAAAAGATCTGTTTAGCTTGCCTCG
natNT2_R	AAAAAAAGATCTCGATTTGCAGGCATTTGCTCGG
NCP1_HindIII	AAAAAAAAGCTTGGAATCACACCGCGAAACTGG
NCP1_XbaI	AAAAAATCTAGAGAAGAACGGTAAGCCTGATCCCG
DAP1_HindIII	AAAAAAAAGCTTGCTGTGCCAGATCAGTGTGGG
DAP1_XbaI	AAAAAATCTAGAGACACCACCGCAGCCACAACC

Figure S1

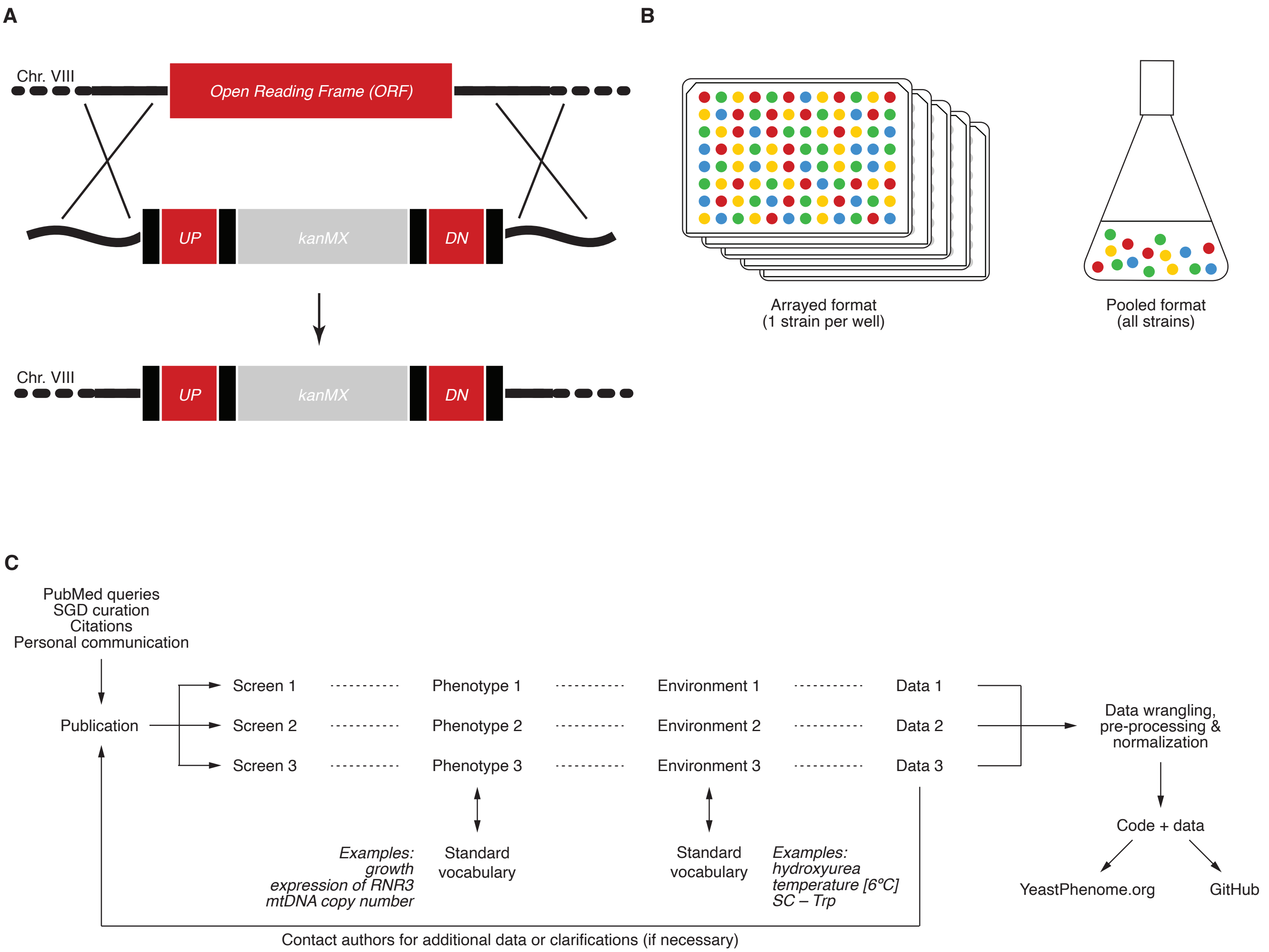

All published screens of the YKO collection were identified, curated, assembled and normalized to enable analysis and integration. (**A**) In the YKO collection, each open reading frame is deleted and replaced via homologous recombination with a selectable marker (kanMX) flanked by locus-specific molecular barcodes (UP and DN), as well as universal sequences that can be used for amplification (black vertical bars). (**B**) Phenotypic screens involving the YKO collection are typically performed in an arrayed or a pooled format. In the arrayed format, each strain is examined independently from other strains by virtue of being grown in a separate well in a 96-well plate and/or as a separate colony on solid media. In the pooled format, all strains are co-cultured together in the same vessel and identified by barcode sequencing or microarray hybridization. (**C**) Publications that report phenotypic screens of the YKO collection were discovered using a comprehensive strategy (Materials & Methods). Each publication was associated with a list of screens and each screen was annotated with a set of standard vocabularies, i.e. lists of standardized terms that describe the measured phenotype (e.g., growth, expression of *RNR3*, mtDNA copy number) and the environment or experimental condition in which the phenotype was measured (e.g., growth medium, exposure to a chemical compound, temperature). Each screen was also associated with the corresponding data, which comprise the list of tested knock-out mutants (whenever available) and the list of phenotypic values for each tested mutant (whenever available). These data were cleaned, harmonized and normalized (Materials & Methods). The original and normalized data, as well as the Python code used for processing, were also stored in a database and a GitHub repository (see “Data and materials availability”).

Figure S2

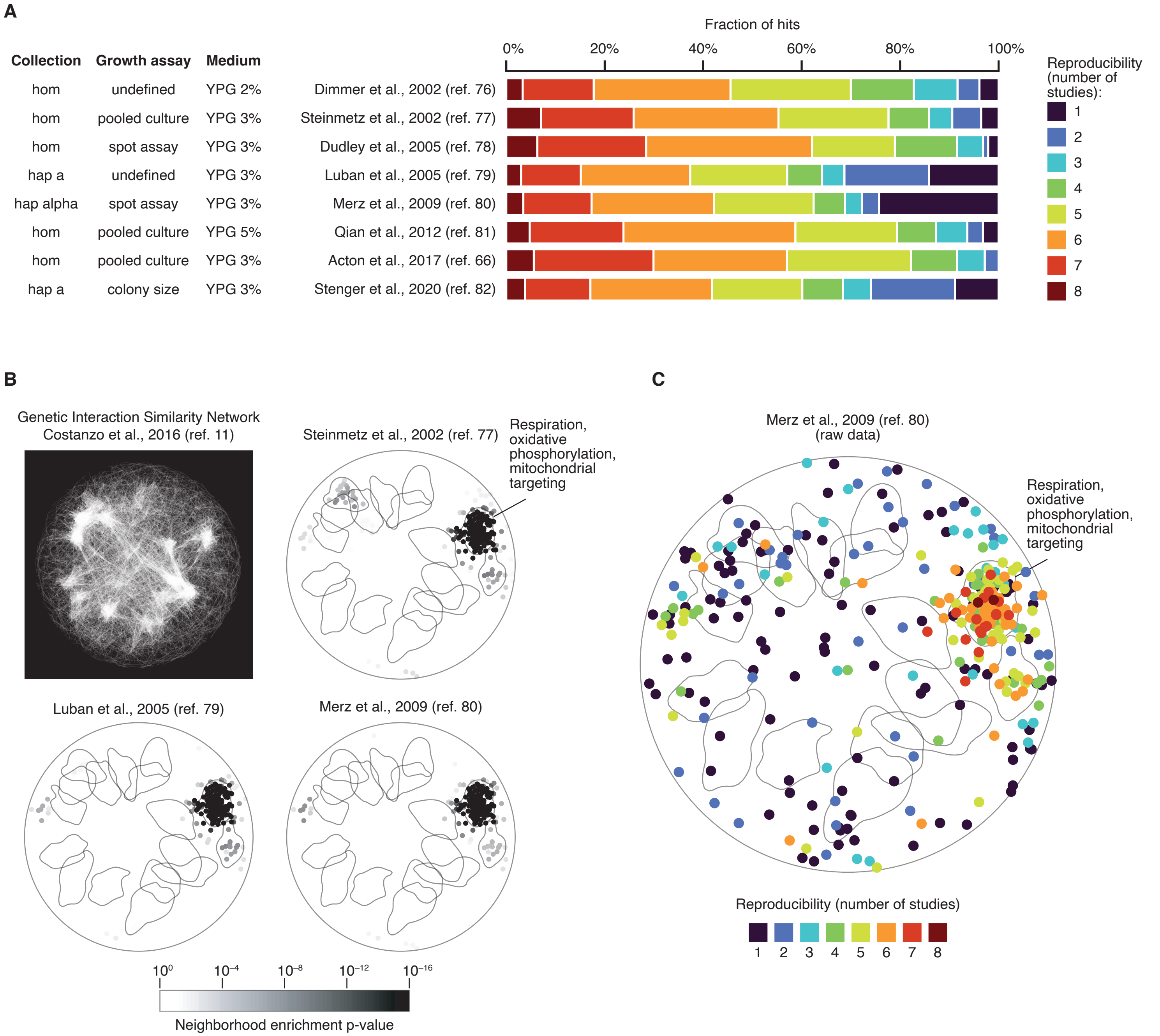

Thanks to its size and meta-data annotations, Yeast Phenome allows to identify similar screens and assess their reproducibility (note S1). (**A**) We identified 8 independent screens of respiratory metabolism (i.e., growth on rich media with glycerol as sole carbon source) using criteria described in table S1. In each screen, "hits" were defined as knock-out mutants with a strong growth defect (NPV < –3) relative to the most typical mutant in that screen (i.e., mode of all phenotypic values). The fractions of hits identified in one screen and reproduced in 0–7 other screens are shown as stacked bars and color-coded. For example, ~4% of hits reported by the first screen (Dimmer et al., 2002 (*76*)) were unique to that screen (black). In contrast, ~18% of hits were reproduced by 6 or 7 other studies (dark red + red). (**B**) The reproducibility of respiration deficiency across the 8 screens was nearly complete when, instead of a gene-by-gene overlap, we compared their SAFE enrichment profiles. A screen's SAFE profile illustrates the statistical association between the identified hits and one or more domains of the genetic interaction similarity network *(11)*. Visual and quantitative comparisons of the SAFE profiles of the 8 screens (3 of which are shown here) demonstrate that, on a functional level, the sets of identified hits are highly similar to one another and consistently associated with respiration, oxidative phosphorylation and mitochondrial targeting functions. (**C**) Reproducible hits are more likely to associate with relevant biological functions than non-reproducible hits. Nodes of the genetic interaction similarity network (B) that correspond to hits from Merz et al., 2009 (*80*) are represented as dots. The color of each dot indicates the total number of screens in which that gene was identified as a hit. Dark red, red, orange and light green colors indicate hits reproduced in at least 5 of the 8 studies. As expected, these hits are concentrated in the network domain associated with respiration, oxidative phosphorylation and mitochondrial targeting. In contrast, black and dark blue dots indicate genes identified in only 1 or 2 screens. These hits are more randomly distributed throughout the network.

Figure S3

A **B**

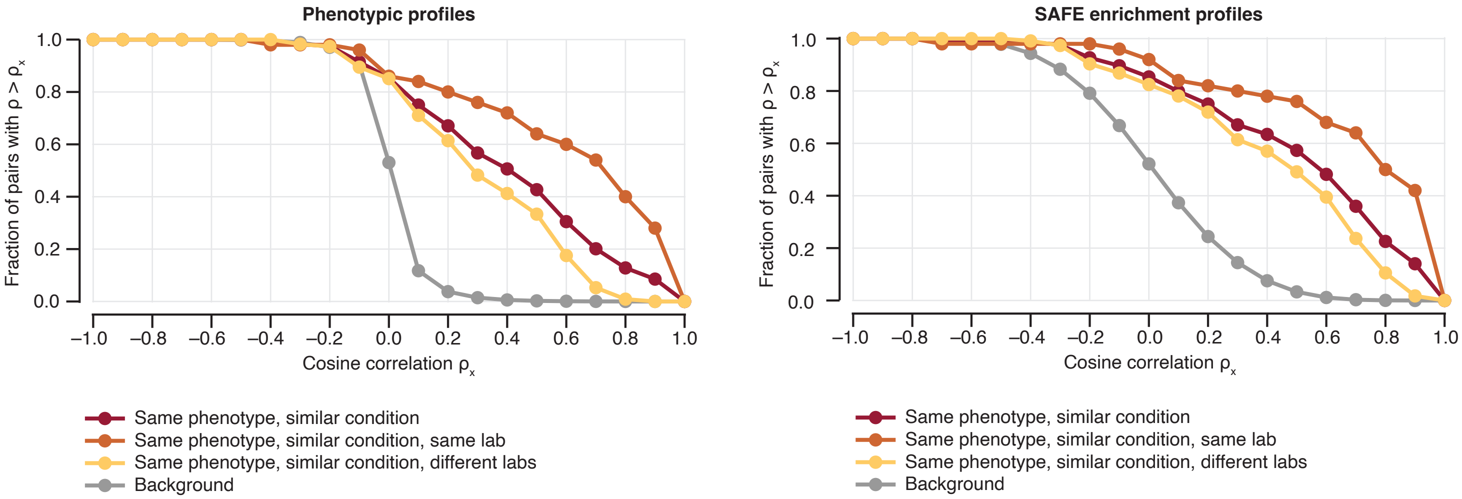

The comparison of 164 pairs of near-replicate screens provides an estimate of screen reproducibility within and between labs. The plots show a cumulative distribution of near-replicate screen-screen similarities computed using phenotypic profiles (**A**) and SAFE enrichment profiles (**B**). Near-replicate screen pairs were defined as screens that have tested the same phenotype under similar experimental conditions (Materials & Methods). Additionally, screens performed by the same lab were analyzed separately from screens performed by different labs. The background distribution corresponds to all screens, regardless of their tested phenotype, condition or lab of origin.

Figure S4

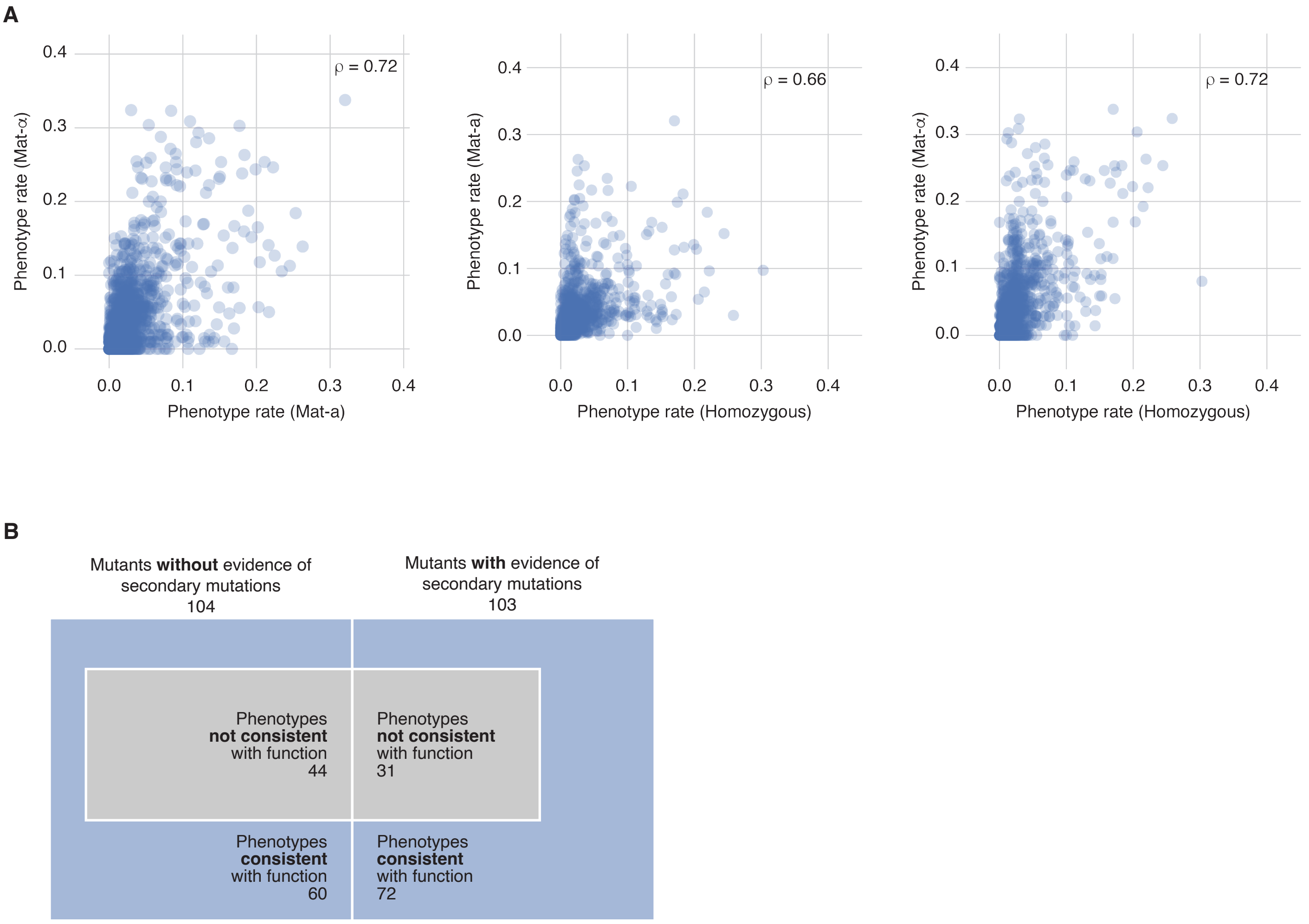

Secondary mutations are unlikely to impact gene-phenotype associations from YKO screens (note S2). (**A**) Phenotype rate is defined as the fraction of screens in which a knock-out mutant shows a strong phenotype (|NPV| > 3). The phenotype rates of Mat-a, Mat-α and homozygous diploid strains mutated for the same genes are generally correlated (cosine correlation ρ = 0.66–0.72), suggesting that secondary mutations are either rare, reoccur frequently in strains lacking the same gene or have relatively little impact on most phenotypes. (**B**) The phenotypic profiles of knock-out mutants with evidence of secondary mutations are more, not less, likely to be consistent with known functions of the knocked-out genes than the phenotypic profiles of mutants without evidence of secondary mutations. Each blue box represents a set of knock-out mutants with (right) and without (left) evidence of secondary mutations (note S2). The grey areas in each box are proportional to the fraction of mutants presenting phenotypes that are inconsistent with the gene's known function.

Figure S5

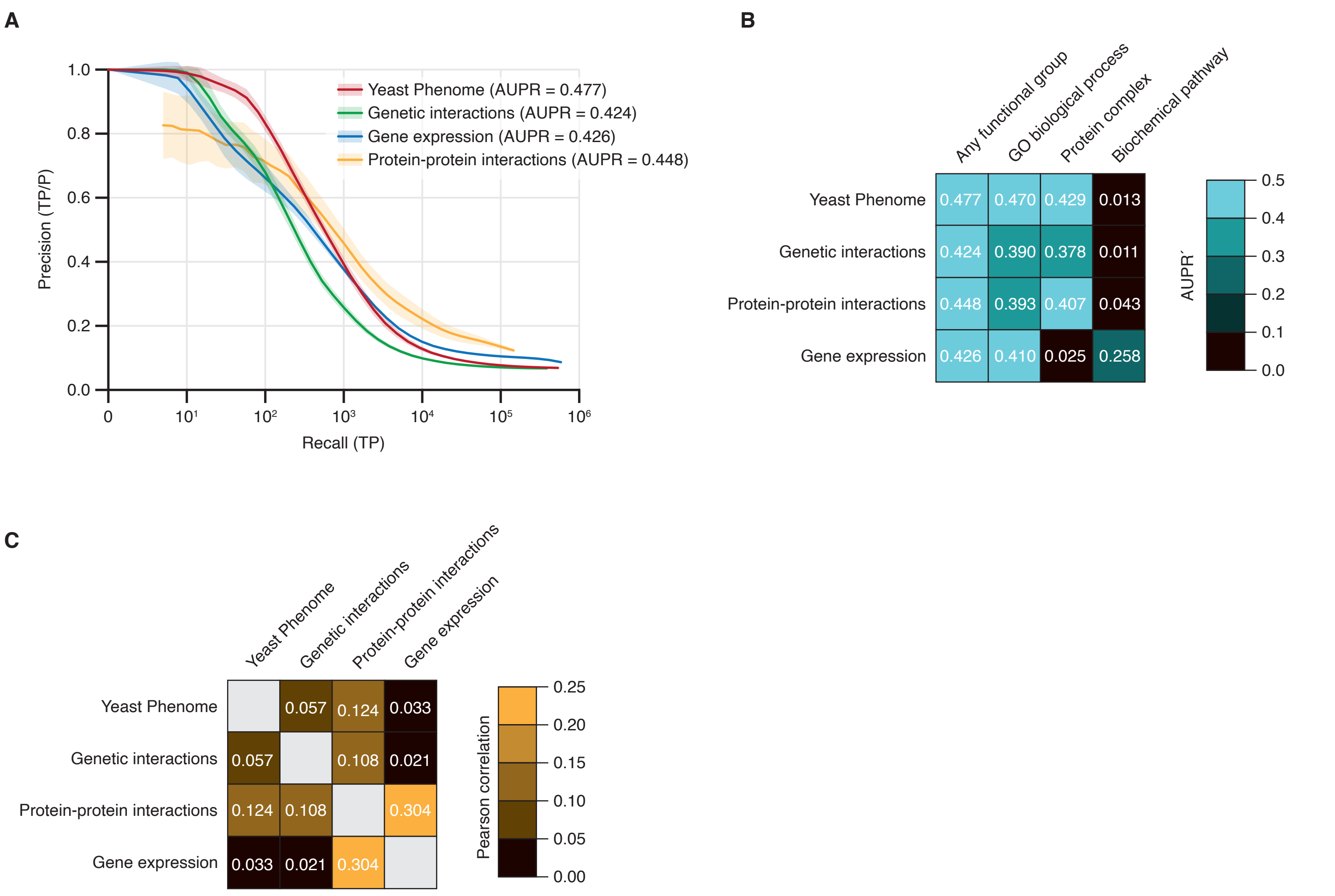

Phenotypic profiles predict functional relationships as accurately as other genome-scale datasets. (**A**) We examined gene-gene similarities using 4 independent data sources: Yeast Phenome, genetic interactions, protein-protein interactions and gene expression. For each dataset, we calculated profile similarities using a bootstrap strategy (20 samples, 1,500 features per sample). In each sample, we ranked gene pairs by their profile similarity (ρ) and computed recall (number of functionally related pairs with $\rho > \alpha$ for decreasing values for α) and precision (the fraction of functionally related pairs among all gene pairs with $\rho > \alpha$ for decreasing values of α). A gene pair was considered functionally related if both genes are co-annotated to the same GO biological process term, protein complex or biochemical pathway. The plot shows the relationship between recall and precision for each dataset. Lines and shaded areas represent the average and standard deviation of precision-recall curves for the 20 samples. Data sources and details about calculating phenotypic similarities, precision-recall and areas under the precision-recall curve (AUPR) are described in Materials & Methods. (**B**) Different types of functional relationships are better predicted by different data types. The heatmap shows areas under the precision-recall curves (AUPRs) computed following the precision-recall analysis described in (A) but using narrower definitions of functional relationship (e.g., only co-annotation to the same protein complex or only co-annotation to the same biochemical pathway). (**C**) Despite an overall consistent performance in functional prediction, we observed little redundancy between data types such that genes correlated in one dataset were generally uncorrelated in others. The heatmap shows Pearson correlation coefficients between gene-gene profile similarity values computed from the 4 different data sources.

Figure S6

A

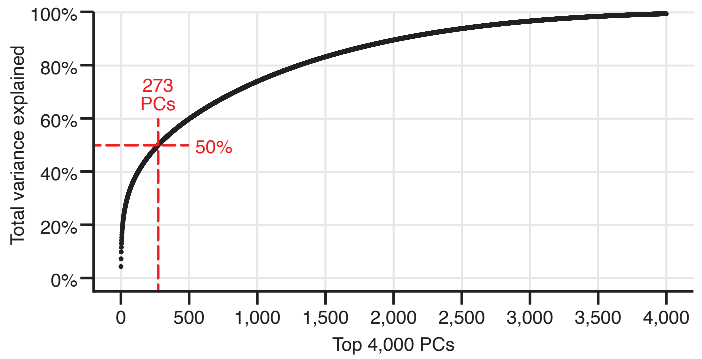

B

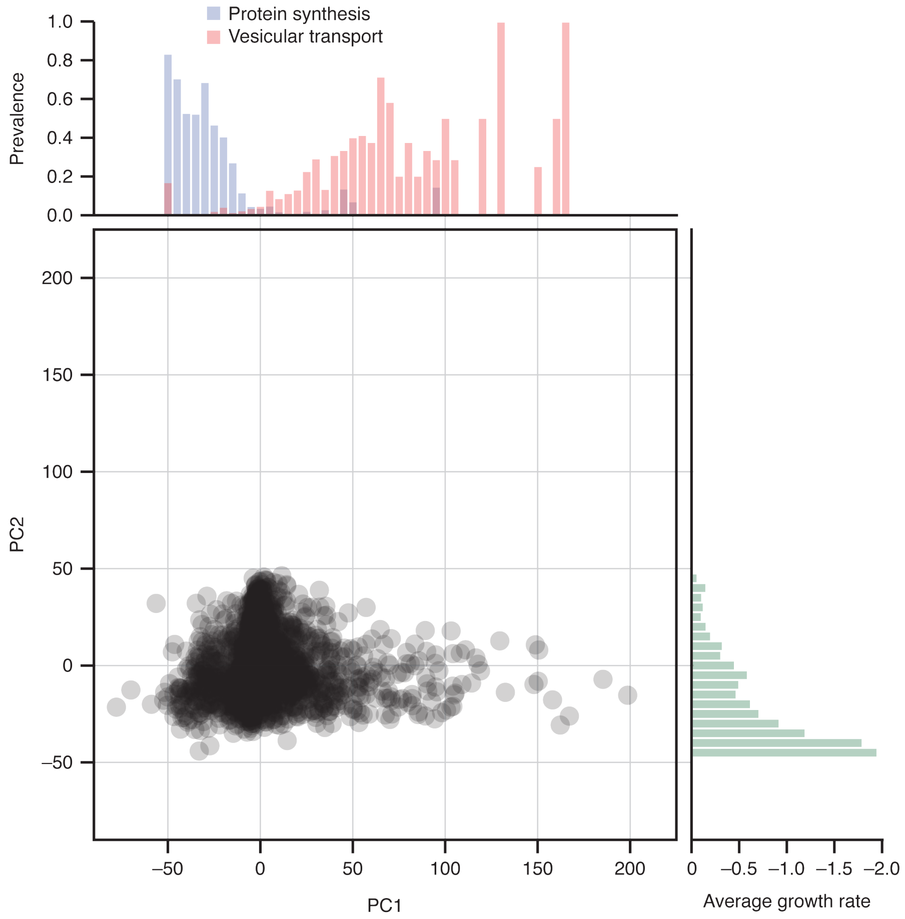

Principal Component Analysis (PCA) of Yeast Phenome data provides an estimate of screen diversity and major axes of variation. (**A**) Cumulative distribution of the phenotypic variation among knock-out mutants explained by the first 4,000 principal components (PCs). The red lines indicate that 50% of phenotypic variance is explained by the first 273 PCs. (**B**) Knock-out mutants projected onto the first 2 PCs of Yeast Phenome data. The bar plots along the x- and y-axis show the association of the 2 PCs with additional gene features. The bar plot on top shows the relative prevalence of genes involved in protein synthesis (blue) and vesicle transport (red) in bins along the PC1 coordinate. The bar plot on the right shows the average mutant growth rate in bins along the PC2 coordinate.

Figure S7

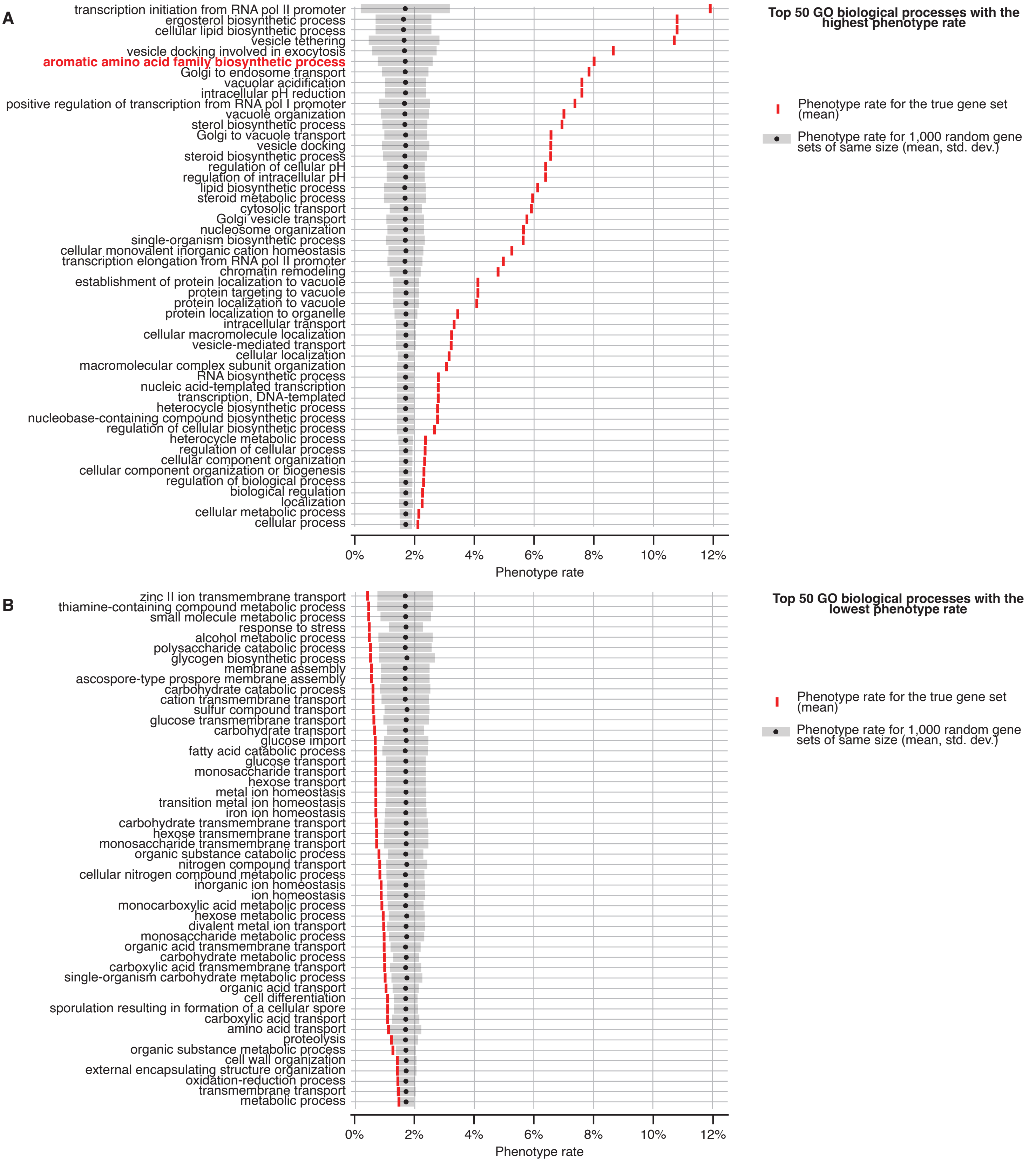

Phenotype rate, defined as the fraction of screens in which a gene shows a strong phenotype (|NPV| > 3), is not uniformly distributed across biological processes. An average phenotype rate was computed for each GO biological process with more than 10 genes represented in Yeast Phenome. The true phenotype rate (P_{true}, red line) was compared to the average and standard deviation of 1,000 randomly sampled gene sets of the same size (P_{random}, black dot and σ_{random}, grey box, respectively). A z-score for each biological process was computed as $(P_{true} - P_{random}) / \sigma_{random}$. The top 50 biological processes with the highest (**A**) and the lowest (**B**) z-scores are shown. The aromatic amino acid family biosynthetic process (highlighted in red) is the only metabolic process with an elevated phenotype rate.

Figure S8

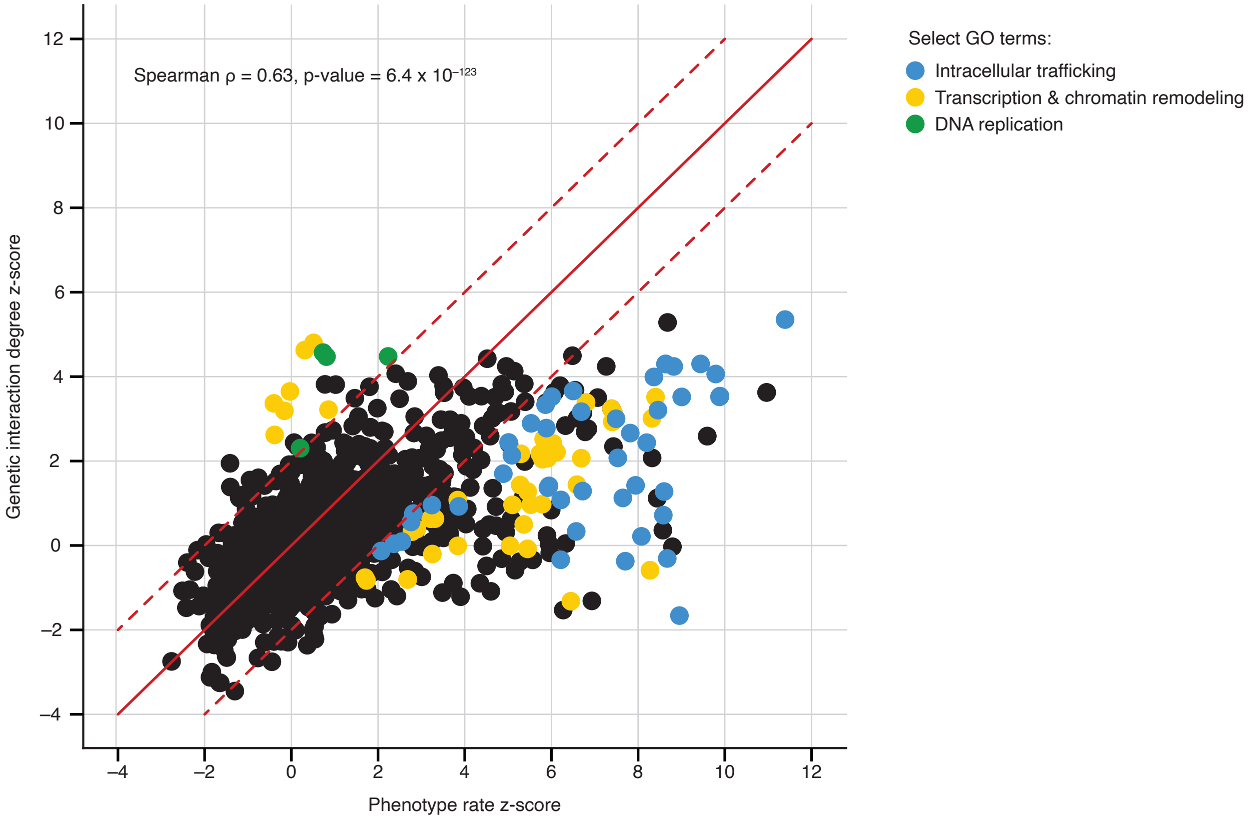

Phenotype rates and genetic interaction degrees for 1,099 GO biological processes are correlated. An average phenotype rate was computed for each process with more than 10 genes represented in Yeast Phenome. A z-score was computed by comparing the true phenotype rate (P_{true}) to the average and standard deviation of 1,000 randomly sampled gene sets of the same size (P_{random}): $Z = (P_{true} - P_{random}) / \sigma_{random}$. The same calculation was performed for genetic itneraction degrees. A scatter-plot of phenotype rate and genetic interaction degree z-scores is shown. The solid red line corresponds to y = x. The dotted red lines correspond to y = x – 2 and y = x + 2. Processes that fall outside of the red dotted lines and are associated with intracellular trafficking, transcription/chromatin remodeling and DNA replication are colored in blue, yellow and green, respectively.

Figure S9

A

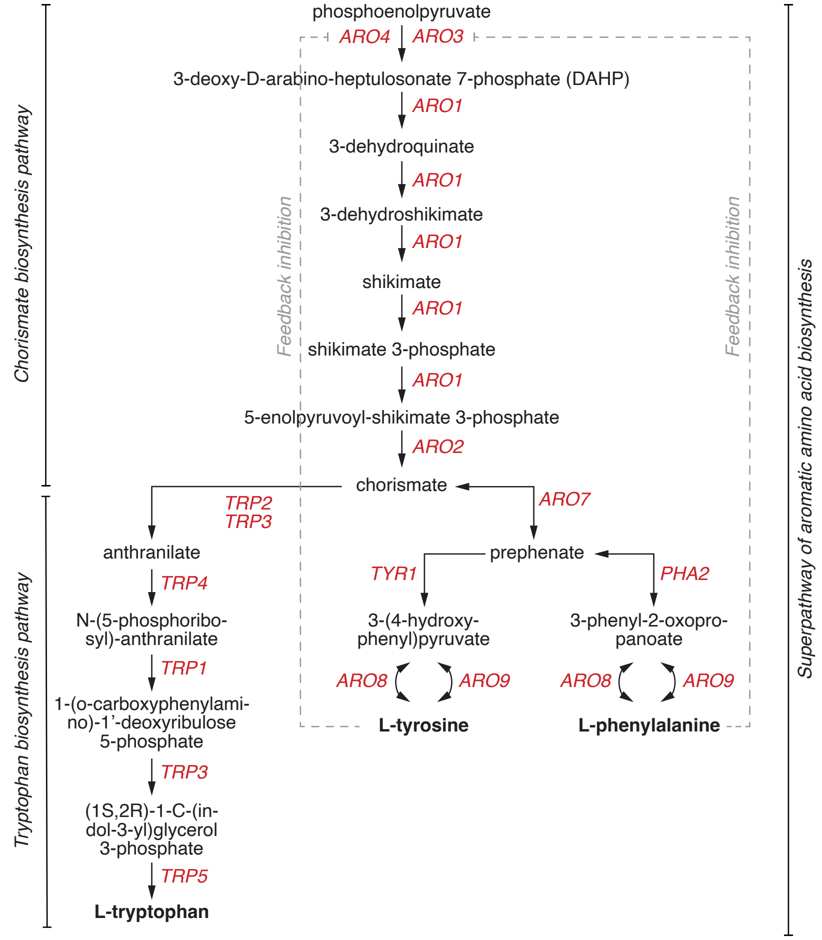

B

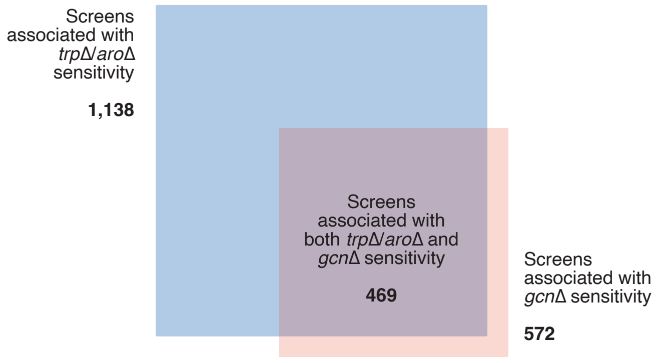

The biosynthesis of tryptophan is unique among all amino acids for its requirement under many chemical stresses. (**A**) The superpathway of aromatic amino acid biosynthesis includes the biosynthesis of tryptophan, tyrosine and phenylalanine, as well as their common precursor chorismate. Genes encoding enzymes required for each reaction are indicated in red. The feedback inhibition of *ARO4* by tyrosine and *ARO3* by phenylalanine are indicated by the grey dashed lines. (**B**) Mutants impaired in the GAAC pathway (*gcn2*Δ, *gcn3*Δ, *gcn4*Δ and *gcn20*Δ) are sensitive to only ~38% of the conditions that cause *trp*Δ/*aro*Δ sensitivity. The Venn diagram shows the overlap between two sets of screens: 1) blue: 1,138 screens where at least 4 of the 8 *trp*Δ/*aro*Δ mutants show impaired growth (NPV < -2); 2) red: 572 screens where at least 2 of the 4 *gcn*Δ mutants show impaired growth (NPV < -2).

Figure S10

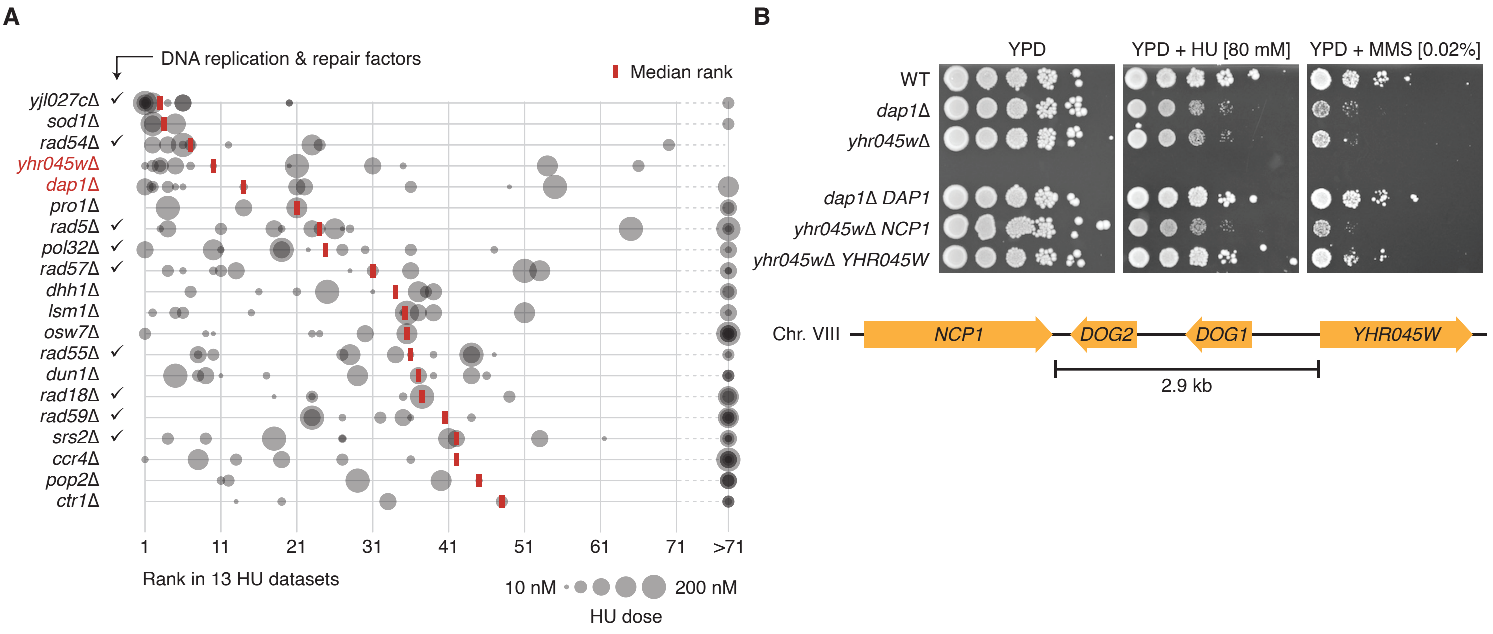

Evidence from Yeast Phenome and validation experiments suggests that Yhr045w works with Dap1 in regulating ergosterol biosynthesis and DNA damage response. (**A**) *yhr045w*Δ and *dap1*Δ are among the top 5 mutants with the highest degree of sensitivity to hydroxyurea (HU). We examined 13 Yeast Phenome screens that tested growth upon exposure to HU at various doses. Mutants were ranked based on their normalized phenotypic values (NPVs) in each screen. The top 20 mutants with the lowest median rank (red line) are shown in the plot. Mutant ranks in all 13 datasets are also shown (grey circles). The size of the circles reflects the HU dose. *yhr045w*Δ and *dap1*Δ are highlighted in red. (**B**) We experimentally confirmed the sensitivity of *dap1*Δ and *yhr045w*Δ to HU and MMS, and verified that the sensitivity is rescued by the expression of plasmid-borne *DAP1* and *YHR045W*, respectively (Materials & Methods). The sensitivity of *yhr045w*Δ cannot be rescued by the expression of *NCP1*, a gene located 2.9 kb upstream of *YHR045W* and involved in ergosterol biosynthesis like *DAP1*. The inability of *NCP1* to complement *yhr045w*Δ phenotypes indicates that these phenotypes are not caused by a neighboring gene effect (NGE).

Figure S11

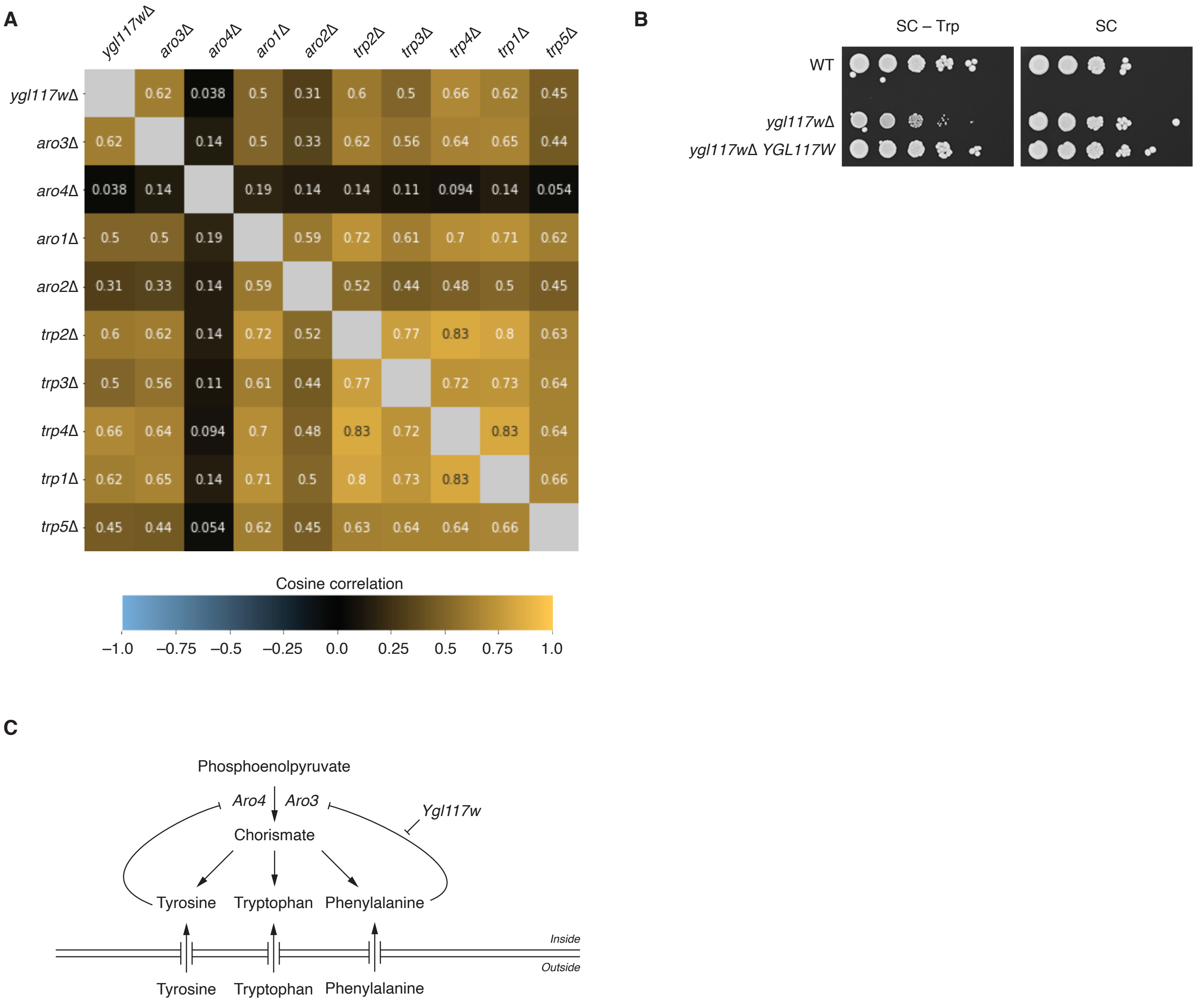

Evidence from Yeast Phenome and validation experiments suggests that Ygl117w is a novel member or regulator of the aromatic amino acid biosynthesis pathway. (**A**) The phenotypic profile of *ygl117wΔ* is as similar to the phenotypic profiles of *trpΔ*/*aroΔ* mutants as they are to one another. Cosine correlations between all pairs of genes were computed using the bootstrap method described in Materials & Methods. (**B**) The growth defect of *ygl117wΔ* on media lacking tryptophan (SC – Trp) is rescued by the expression of a plasmid-borne *YGL117W* (Materials & Methods). (**C**) A model describing the potential role of Ygl117w in the aromatic amino acid biosynthesis pathway. Data in the literature, Yeast Phenome data and our own validation experiments are consistent with the hypothesis that Ygl117w negatively regulates the ability of phenylalanine to feedback-inhibit the DAHP activity of Aro3.

Figure S12

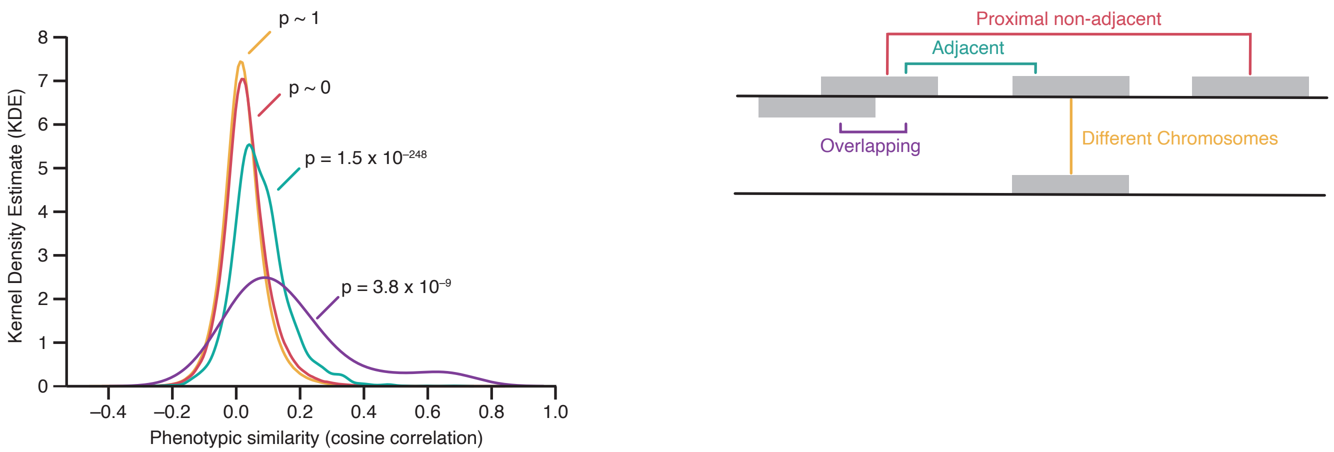

The phenotypic similarities of overlapping, immediately adjacent and proximal non-adjacent gene pairs are significantly higher than expected by random chance. A schematic representation of different classes of gene pairs is shown on the right. The distributions of phenotypic similarities for overlapping gene pairs (purple), adjacent gene pairs (cyan), proximal non-adjacent gene pairs (red) and gene pairs located on different chromosomes (yellow) were compared to the overall distribution of all phenotypic similarities (excluding overlapping gene pairs). The p-values for the corresponding 2-sample Kolmogorov-Smirnov tests are reported.

Figure S13

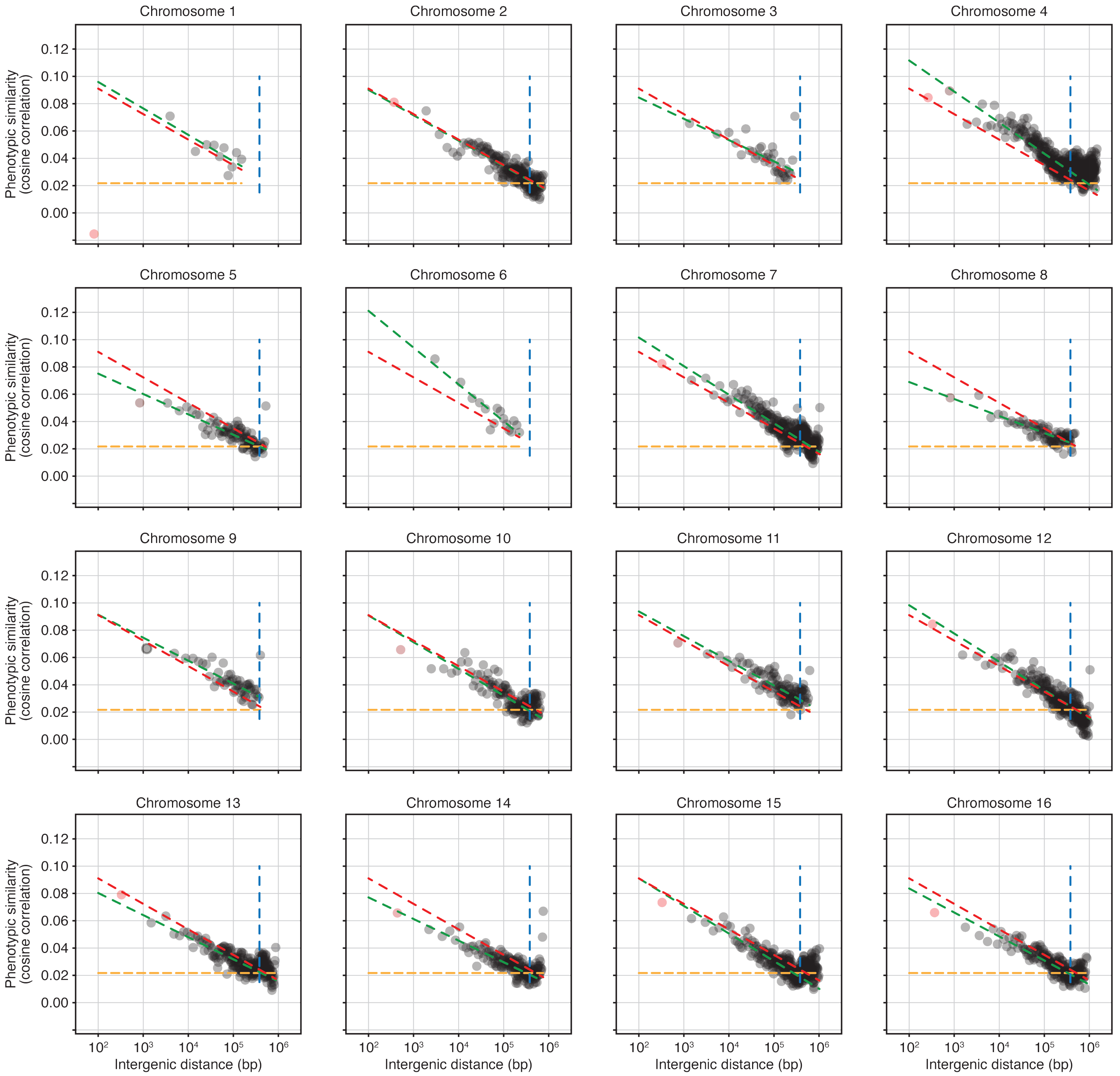

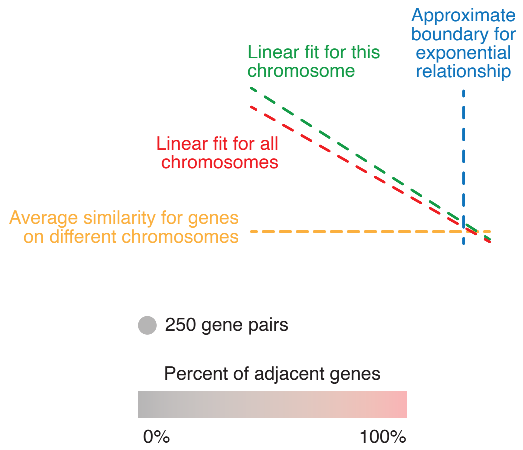

The relationship between phenotypic similarity and chromosomal proximity is consistent across all chromosomes examined independently. Gene pairs located on each chromosome were sorted by their intergenic distance and subdivided into groups of 250 pairs. In each group, the average intergenic distance and average phenotypic similarity were computed and plotted on the x and y-axis, respectively. Distance was plotted on a $\log_{10}$ scale. The color of each point indicates the fraction of immediately adjacent genes in the group. The yellow line indicates the average phenotypic similarity for gene pairs located on different chromosomes. The blue line indicates the approximate boundary of the exponential relationship estimated from all chromosomes (380 kb; Fig. 5; Materials & Methods). The green line indicates the linear fit between $\log_{10}$ intergenic distance and phenotypic similarity for all points within the estimated distance boundary (left of the blue line) on each chromosome. As a reference, the red line indicates the same linear fit estimated from all chromosomes (Fig. 5).

Figure S14

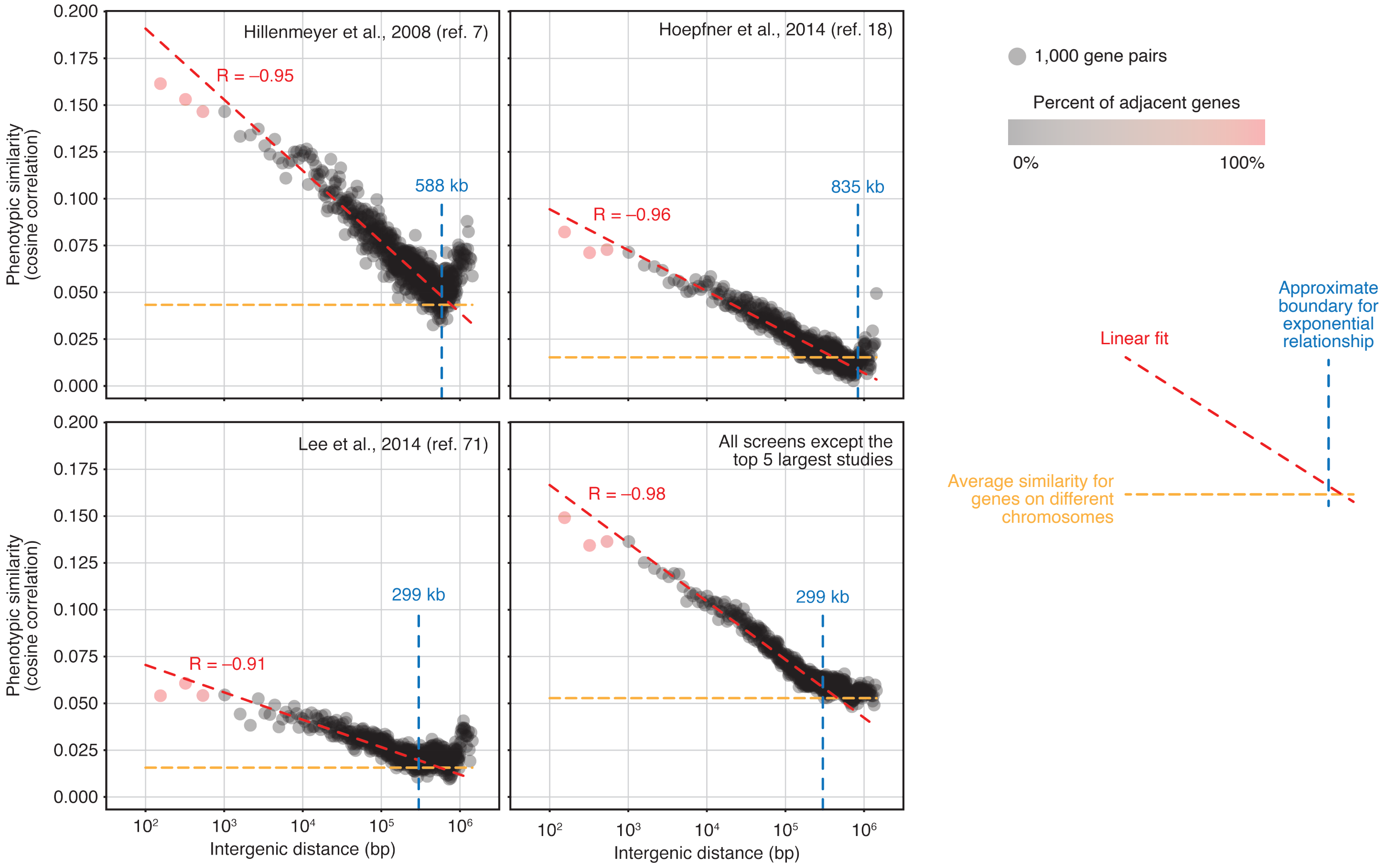

The relationship between phenotypic similarity and chromosomal proximity is consistent across several independent subsets of Yeast Phenome data. Phenotypic similarity was computed using only screens from 3 large studies (Hillenmeyer et al., 2008 (*7*); Hoepfner et al., 2014 (*18*); Lee et al., 2014 (*71*)), as well as all screens excluding the top 5 largest chemo-genomics datasets (the top 3 plus Parsons et al., 2006 (*6*); Ericson et al., 2008 (*72*)). Gene pairs located on the same chromosome were sorted by their intergenic distance and subdivided into groups of 1,000 pairs. In each group, the average intergenic distance and average phenotypic similarity were computed and plotted on the x and y-axis, respectively. Distance was plotted on a $\log_{10}$ scale. The color of each point indicates the fraction of immediately adjacent genes in the group. The yellow line indicates the average phenotypic similarity for gene pairs located on different chromosomes. The blue line indicates the approximate boundary of the exponential relationship estimated from each subset (Materials & Methods). The red line indicates the linear fit between $\log_{10}$ intergenic distance and phenotypic similarity for all points within the estimated distance boundary (left of the blue line).

Figure S15

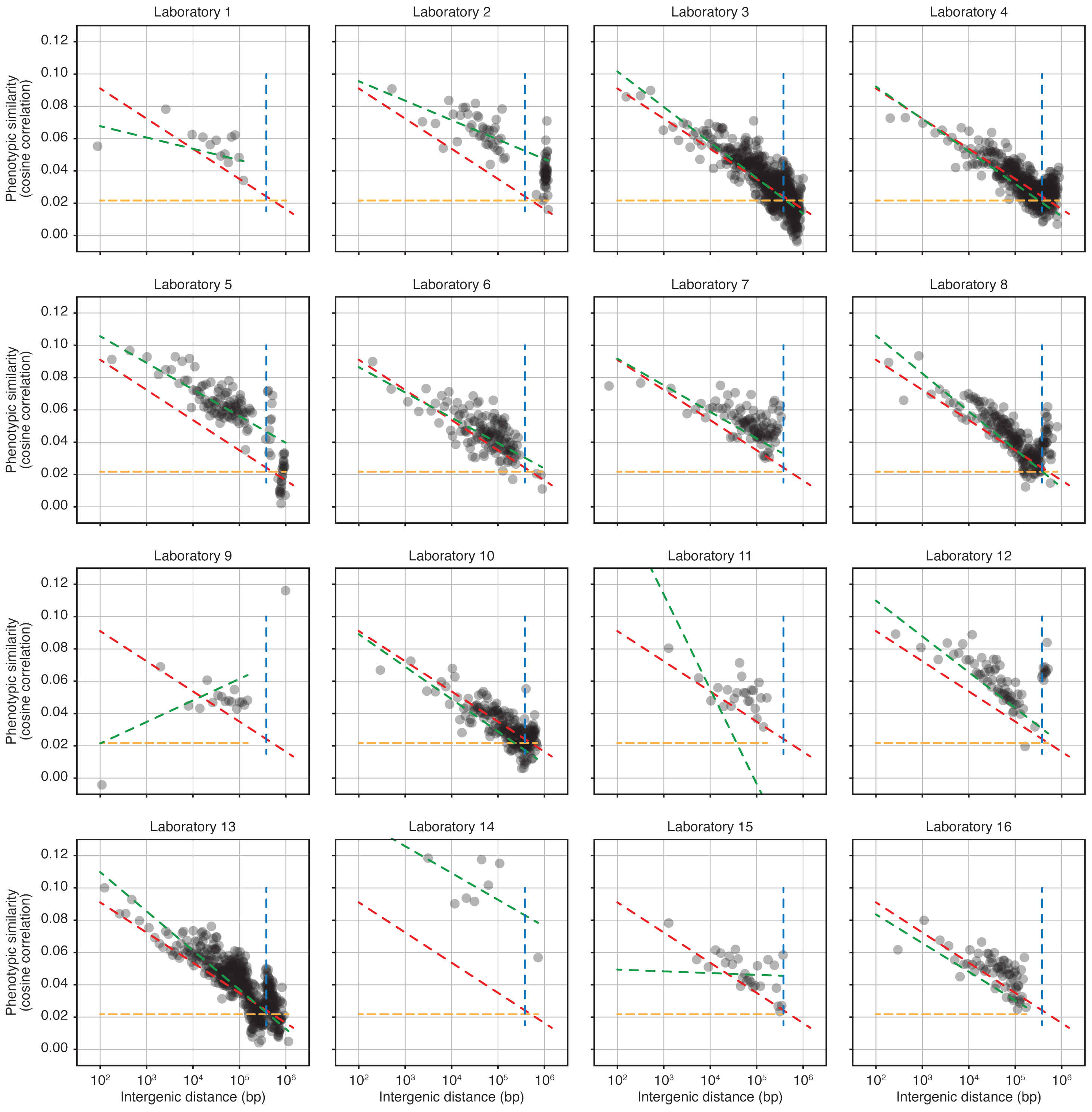

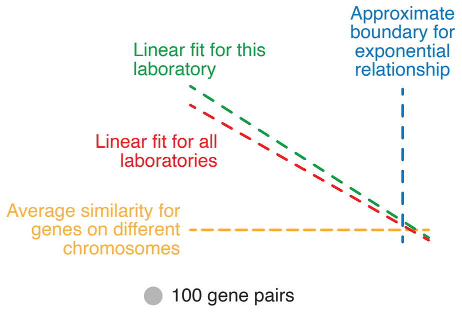

The relationship between phenotypic similarity and chromosomal proximity is consistent across all sets of strains constructed by the same laboratory. Gene pairs located on each chromosome were sorted by their intergenic distance and subdivided into groups of 100 pairs. In each group, the average intergenic distance and average phenotypic similarity were computed and plotted on the x and y-axis, respectively. Distance was plotted on a $\log_{10}$ scale. The yellow line indicates the average phenotypic similarity for gene pairs located on different chromosomes. The blue line indicates the approximate boundary of the exponential relationship estimated from all gene pairs (380 kb; Fig. 5; Materials & Methods). The green line indicates the linear fit between $\log_{10}$ intergenic distance and phenotypic similarity for all gene pairs within the estimated distance boundary (left of the blue line) constructed by a given laboratory. As a reference, the red line indicates the same linear fit estimated from all gene pairs (Fig. 5). Despite the fact that strains constructed by Laboratory 14 have a consistently higher phenotypic similarity (see main text), they still show an exponential relationship with intergenic distance.

Figure S16

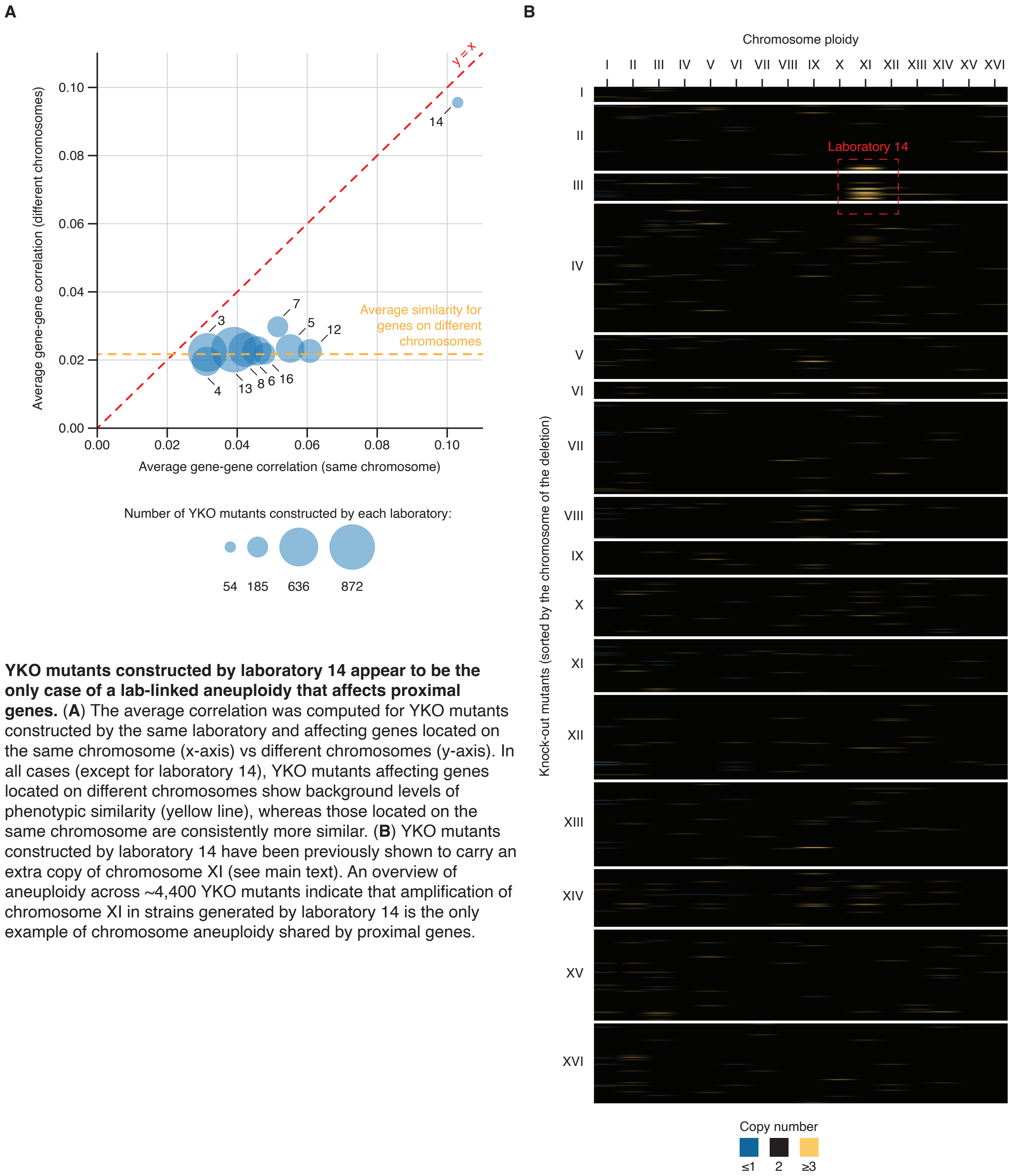

YKO mutants constructed by laboratory 14 appear to be the only case of a lab-linked aneuploidy that affects proximal genes. (**A**) The average correlation was computed for YKO mutants constructed by the same laboratory and affecting genes located on the same chromosome (x-axis) vs different chromosomes (y-axis). In all cases (except for laboratory 14), YKO mutants affecting genes located on different chromosomes show background levels of phenotypic similarity (yellow line), whereas those located on the same chromosome are consistently more similar. (**B**) YKO mutants constructed by laboratory 14 have been previously shown to carry an extra copy of chromosome XI (see main text). An overview of aneuploidy across ~4,400 YKO mutants indicate that amplification of chromosome XI in strains generated by laboratory 14 is the only example of chromosome aneuploidy shared by proximal genes.

Figure S17

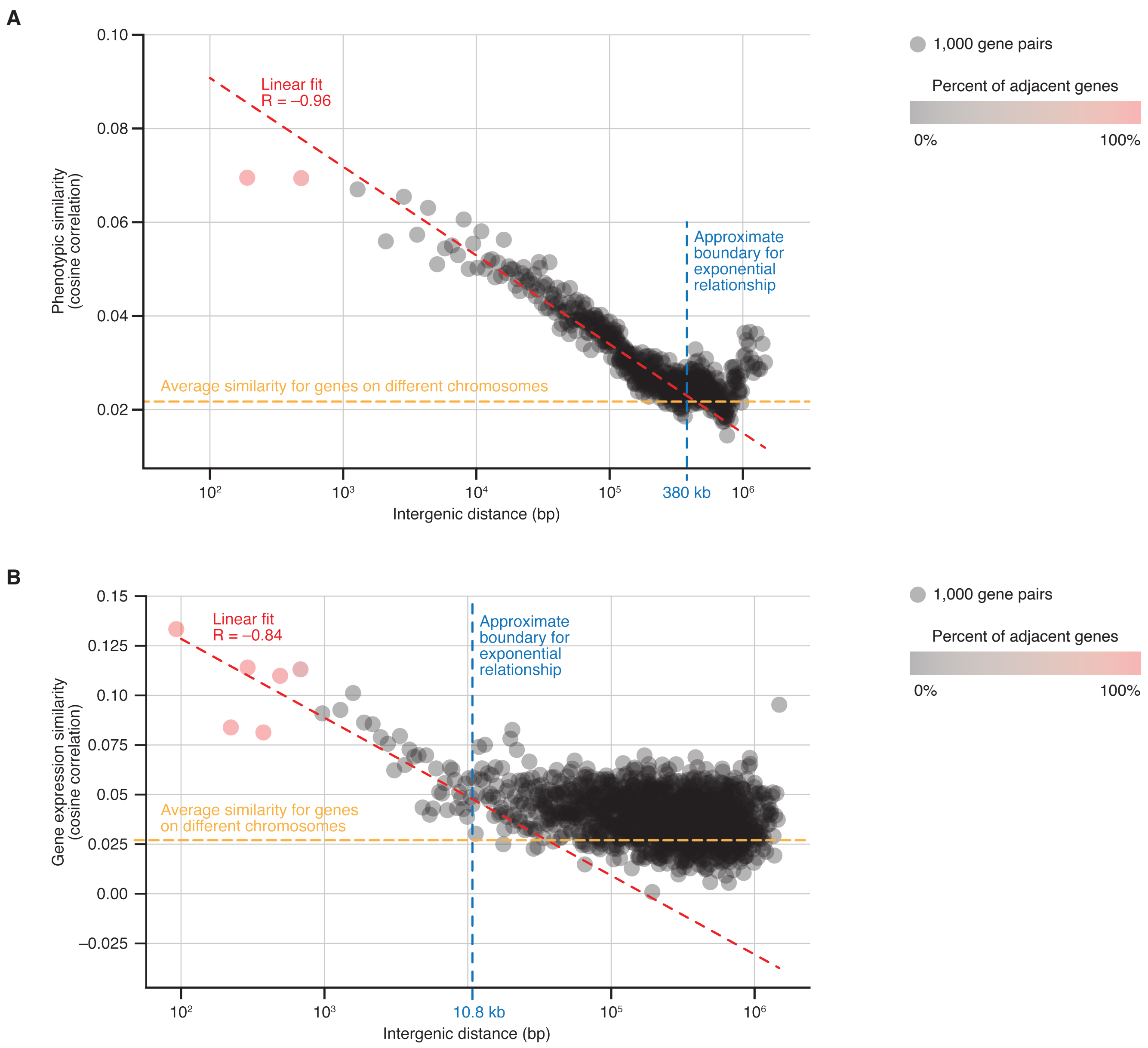

The relationship between phenotypic similarity and chromosomal proximity is not explained by known cases of functional co-clustering. (**A**) The relationship persists if we exclude all gene pairs with prior evidence of functional co-clustering (Materials & Methods): members of the same protein complex, metabolic pathway, moderately specific GO biological process term; gene co-expressed or co-regulated by the same transcription factor; paralogous gene pairs. Gene pairs located on the same chromosome were sorted by their intergenic distance and subdivided into groups of 1,000 pairs. In each group, the average intergenic distance and average phenotypic similarity were computed and plotted on the x and y-axis, respectively. Distance was plotted on a log_{10} scale. The color of each point indicates the fraction of immediately adjacent genes in the group. The yellow line indicates the average phenotypic similarity for gene pairs located on different chromosomes. The blue line indicates the approximate boundary of the exponential relationship estimated from each subset (Materials & Methods). The red line indicates the linear fit between log_{10} intergenic distance and phenotypic similarity for all points within the estimated distance boundary (left of the blue line). (**B**) Similarity of gene expression across multiple experimental conditions (*37*) is also related to chromosomal proximity. However, this relationship has a much shorter range (10.8 kb) than phenotypic similarity (380 kb). Gene pairs located on the same chromosome were sorted by their intergenic distance and subdivided into groups of 1,000 pairs. In each group, the average intergenic distance and average gene co-expression (measured by cosine similarity) were computed and plotted on the x and y-axis, respectively. Distance was plotted on a log_{10} scale. The color of each point indicates the fraction of immediately adjacent genes in the group. The yellow line indicates the average phenotypic similarity for gene pairs located on different chromosomes. The blue line indicates the approximate boundary of the exponential relationship estimated from each subset (Materials & Methods). The red line indicates the linear fit between log_{10} intergenic distance and phenotypic similarity for all points within the estimated distance boundary (left of the blue line).

Figure S18

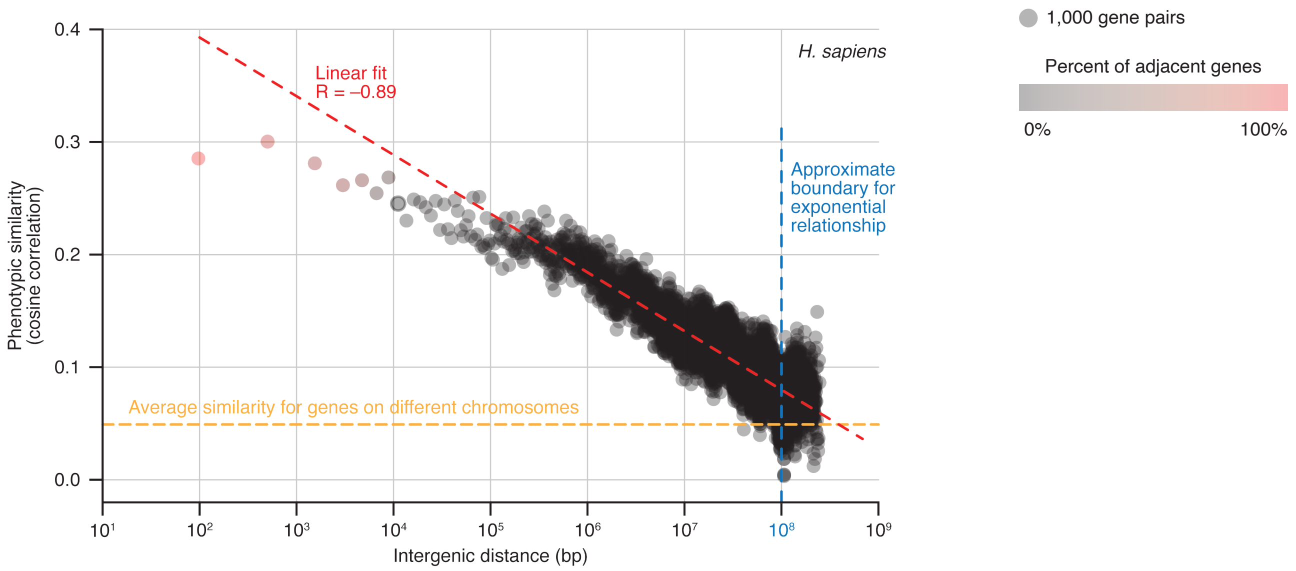

The relationship between phenotypic similarity (co-essentiality) and chromosomal proximity among human genes persists if we exclude amplified genes. Genes with reported evidence of copy number amplification ($n > 4$) in at least 2 cancer cell lines were excluded from the analysis (Materials & Methods). Gene pairs located on the same chromosome were sorted by their intergenic distance and subdivided into groups of 1,000 pairs. In each group, the average intergenic distance and average phenotypic similarity were computed and plotted on the x and y-axis, respectively. Distance was plotted on a $\log_{10}$ scale. The color of each point indicates the fraction of immediately adjacent genes in the group. The yellow line indicates the average phenotypic similarity for gene pairs located on different chromosomes. The blue line indicates the approximate boundary of the exponential relationship estimated from each subset. The red line indicates the linear fit between $\log_{10}$ intergenic distance and phenotypic similarity for all points within the estimated distance boundary (left of the blue line).

REFERENCES AND NOTES

1. E. Melamud, D. Leland Taylor, A. Sethi, M. Cule, A. Baryshnikova, D. Saleheen, N. van Bruggen, G. A. FitzGerald, The promise and reality of therapeutic discovery from large cohorts. *J. Clin. Investig.* **130**, 575–581 (2020).

2. G. Giaever, C. Nislow, The yeast deletion collection: A decade of functional genomics. *Genetics* **197**, 451–465 (2014).

3. G. Giaever, A. M. Chu, L. Ni, C. Connelly, L. Riles, S. Véronneau, S. Dow, A. Lucau-Danila, K. Anderson, B. André, A. P. Arkin, A. Astromoff, M. El-Bakkoury, R. Bangham, R. Benito, S. Brachat, S. Campanaro, M. Curtiss, K. Davis, A. Deutschbauer, K.-D. Entian, P. Flaherty, F. Foury, D. J. Garfinkel, M. Gerstein, D. Gotte, U. Güldener, J. H. Hegemann, S. Hempel, Z. Herman, D. F. Jaramillo, D. E. Kelly, S. L. Kelly, P. Kötter, D. LaBonte, D. C. Lamb, N. Lan, H. Liang, H. Liao, L. Liu, C. Luo, M. Lussier, R. Mao, P. Menard, S. L. Ooi, J. L. Revuelta, C. J. Roberts, M. Rose, P. Ross-Macdonald, B. Scherens, G. Schimmack, B. Shafer, D. D. Shoemaker, S. Sookhai-Mahadeo, R. K. Storms, J. N. Strathern, G. Valle, M. Voet, G. Volckaert, C.-Y. Wang, T. R. Ward, J. Wilhelmy, E. A. Winzeler, Y. Yang, G. Yen, E. Youngman, K. Yu, H. Bussey, J. D. Boeke, M. Snyder, P. Philippsen, R. W. Davis, M. Johnston, Functional profiling of the Saccharomyces cerevisiae genome. *Nature* **418**, 387–391 (2002).

4. P. Kemmeren, K. Sameith, L. A. L. van de Pasch, J. J. Benschop, T. L. Lenstra, T. Margaritis, E. O'Duibhir, E. Apweiler, S. van Wageningen, C. W. Ko, S. van Heesch, M. M. Kashani, G. Ampatziadis-Michailidis, M. O. Brok, N. A. C. H. Brabers, A. J. Miles, D. Bouwmeester, S. R. van Hooff, H. van Bakel, E. Sluiters, L. V. Bakker, B. Snel, P. Lijnzaad, D. van Leenen, M. J. A. Groot Koerkamp, F. C. P. Holstege, Large-scale genetic perturbations reveal regulatory networks and an abundance of gene-specific repressors. *Cell* **157**, 740–752 (2014).

5. A. Baryshnikova, Systematic functional annotation and visualization of biological networks. *Cell Syst.* **2**, 412–421 (2016).

6. A. B. Parsons, A. Lopez, I. E. Givoni, D. E. Williams, C. A. Gray, J. Porter, G. Chua, R. Sopko, R. L. Brost, C.-H. Ho, J. Wang, T. Ketela, C. Brenner, J. A. Brill, G. E. Fernandez, T. C. Lorenz, G. S. Payne, S. Ishihara, Y. Ohya, B. Andrews, T. R. Hughes, B. J. Frey, T. R. Graham, R. J. Andersen, C. Boone,

Exploring the mode-of-action of bioactive compounds by chemical-genetic profiling in yeast. *Cell* **126**, 611–625 (2006).

7. M. E. Hillenmeyer, E. Fung, J. Wildenhain, S. E. Pierce, S. Hoon, W. Lee, M. Proctor, R. P. St Onge, M. Tyers, D. Koller, R. B. Altman, R. W. Davis, C. Nislow, G. Giaever, The chemical genomic portrait of yeast: Uncovering a phenotype for all genes. *Science* **320**, 362–365 (2008).

8. A. Zakrzewska, G. van Eikenhorst, J. E. C. Burggraaff, D. J. Vis, H. Hoefsloot, D. Delneri, S. G. Oliver, S. Brul, G. J. Smits, Genome-wide analysis of yeast stress survival and tolerance acquisition to analyze the central trade-off between growth rate and cellular robustness. *Mol. Biol. Cell* **22**, 4435–4446 (2011).

9. M. Costanzo, A. Baryshnikova, J. Bellay, Y. Kim, E. D. Spear, C. S. Sevier, H. Ding, J. L. Y. Koh, K. Toufighi, S. Mostafavi, J. Prinz, R. P. St Onge, B. VanderSluis, T. Makhnevych, F. J. Vizeacoumar, S. Alizadeh, S. Bahr, R. L. Brost, Y. Chen, M. Cokol, R. Deshpande, Z. Li, Z.-Y. Lin, W. Liang, M. Marback, J. Paw, B.-J. San Luis, E. Shuteriqi, A. H. Y. Tong, N. van Dyk, I. M. Wallace, J. A. Whitney, M. T. Weirauch, G. Zhong, H. Zhu, W. A. Houry, M. Brudno, S. Ragibizadeh, B. Papp, C. Pál, F. P. Roth, G. Giaever, C. Nislow, O. G. Troyanskaya, H. Bussey, G. D. Bader, A.-C. Gingras, Q. D. Morris, P. M. Kim, C. A. Kaiser, C. L. Myers, B. J. Andrews, C. Boone, The genetic landscape of a cell. *Science* **327**, 425–431 (2010).

10. T. F. Cooper, E. A. Ostrowski, M. Travisano, A negative relationship between mutation pleiotropy and fitness effect in yeast. *Evolution* **61**, 1495–1499 (2007).

11. M. Costanzo, B. VanderSluis, E. N. Koch, A. Baryshnikova, C. Pons, G. Tan, W. Wang, M. Usaj, J. Hanchard, S. D. Lee, V. Pelechano, E. B. Styles, M. Billmann, J. van Leeuwen, N. van Dyk, Z.-Y. Lin, E. Kuzmin, J. Nelson, J. S. Piotrowski, T. Srikumar, S. Bahr, Y. Chen, R. Deshpande, C. F. Kurat, S. C. Li, Z. Li, M. M. Usaj, H. Okada, N. Pascoe, B.-J. San Luis, S. Sharifpoor, E. Shuteriqi, S. W. Simpkins, J. Snider, H. G. Suresh, Y. Tan, H. Zhu, N. Malod-Dognin, V. Janjic, N. Przulj, O. G. Troyanskaya, I. Stagljar, T. Xia, Y. Ohya, A.-C. Gingras, B. Raught, M. Boutros, L. M. Steinmetz, C. L. Moore, A. P. Rosebrock, A. A. Caudy, C. L. Myers, B. Andrews, C. Boone, A global genetic interaction network maps a wiring diagram of cellular function. *Science* **353**, eaaf1420 (2016).

12. F. Abe, K. Horikoshi, Tryptophan permease gene TAT2 confers high-pressure growth in Saccharomyces cerevisiae. *Mol. Cell. Biol.* **20**, 8093–8102 (2000).

13. E. M. Cornett, M. Novitch, A. D. Kaye, V. Kata, A. M. Kaye, Medication-induced tardive dyskinesia: A review and update. *Ochsner J.* **17**, 162–174 (2017).

14. L. Kozell, R. Sandyk, G. C. Wagner, H. Fisher, The effects of L-tryptophan on haloperidol-induced movement disorder in the rat. *Life Sci.* **41**, 1739–1744 (1987).

15. C. M. Tanner, F. Kamel, G. W. Ross, J. A. Hoppin, S. M. Goldman, M. Korell, C. Marras, G. S. Bhudhikanok, M. Kasten, A. R. Chade, K. Comyns, M. B. Richards, C. Meng, B. Priestley, H. H. Fernandez, F. Cambi, D. M. Umbach, A. Blair, D. P. Sandler, J. W. Langston, Rotenone, paraquat, and Parkinson's disease. *Environ. Health Perspect.* **119**, 866–872 (2011).

16. Y. Wang, S. Chen, J. Tan, Y. Gao, H. Yan, Y. Liu, S. Yi, Z. Xiao, H. Wu, Tryptophan in the diet ameliorates motor deficits in a rotenone-induced rat Parkinson's disease model via activating the aromatic hydrocarbon receptor pathway. *Brain Behav.* **11**, e2226 (2021).

17. A. G. Hinnebusch, Translational regulation of GCN_4 and the general amino acid control of yeast. *Annu. Rev. Microbiol.* **59**, 407–450 (2005).

18. D. Hoepfner, S. B. Helliwell, H. Sadlish, S. Schuierer, I. Filipuzzi, S. Brachat, B. Bhullar, U. Plikat, Y. Abraham, M. Altorfer, T. Aust, L. Baeriswyl, R. Cerino, L. Chang, D. Estoppey, J. Eichenberger, M. Frederiksen, N. Hartmann, A. Hohendahl, B. Knapp, P. Krastel, N. Melin, F. Nigsch, E. J. Oakeley, V. Petitjean, F. Petersen, R. Riedl, E. K. Schmitt, F. Staedtler, C. Studer, J. A. Tallarico, S. Wetzel, M. C. Fishman, J. A. Porter, N. R. Movva, High-resolution chemical dissection of a model eukaryote reveals targets, pathways and gene functions. *Microbiol. Res.* **169**, 107–120 (2014).

19. M. C. Jonikas, S. R. Collins, V. Denic, E. Oh, E. M. Quan, V. Schmid, J. Weibezahn, B. Schwappach, P. Walter, J. S. Weissman, M. Schuldiner, Comprehensive characterization of genes required for protein folding in the endoplasmic reticulum. *Science* **323**, 1693–1697 (2009).

20. J. M. Cherry, E. L. Hong, C. Amundsen, R. Balakrishnan, G. Binkley, E. T. Chan, K. R. Christie, M. C. Costanzo, S. S. Dwight, S. R. Engel, D. G. Fisk, J. E. Hirschman, B. C. Hitz, K. Karra, C. J. Krieger,

S. R. Miyasato, R. S. Nash, J. Park, M. S. Skrzypek, M. Simison, S. Weng, E. D. Wong, Saccharomyces Genome database: The genomics resource of budding yeast. *Nucleic Acids Res.* **40**, D700–D705 (2012).

21. W.-K. Huh, J. V. Falvo, L. C. Gerke, A. S. Carroll, R. W. Howson, J. S. Weissman, E. K. O'Shea, Global analysis of protein localization in budding yeast. *Nature* **425**, 686–691 (2003).

22. K. Kehrein, R. Schilling, B. V. Möller-Hergt, C. A. Wurm, S. Jakobs, T. Lamkemeyer, T. Langer, M. Ott, Organization of mitochondrial gene expression in two distinct ribosome-containing assemblies. *Cell Rep.* **10**, 843–853 (2015).

23. R. A. Hand, N. Jia, M. Bard, R. J. Craven, Saccharomyces cerevisiae Dap1p, a novel DNA damage response protein related to the mammalian membrane-associated progesterone receptor. *Eukaryot. Cell* **2**, 306–317 (2003).

24. J. C. Mallory, G. Crudden, B. L. Johnson, C. Mo, C. A. Pierson, M. Bard, R. J. Craven, Dap1p, a heme-binding protein that regulates the cytochrome P450 protein Erg11p/Cyp51p in Saccharomyces cerevisiae. *Mol. Cell. Biol.* **25**, 1669–1679 (2005).

25. F. Puddu, M. Herzog, A. Selivanova, S. Wang, J. Zhu, S. Klein-Lavi, M. Gordon, R. Meirman, G. Millan-Zambrano, I. Ayestaran, I. Salguero, R. Sharan, R. Li, M. Kupiec, S. P. Jackson, Genome architecture and stability in the Saccharomyces cerevisiae knockout collection. *Nature* **573**, 416–420 (2019).

26. S. R. Hackett, E. A. Baltz, M. Coram, B. J. Wranik, G. Kim, A. Baker, M. Fan, D. G. Hendrickson, M. Berndl, R. Scott McIsaac, Learning causal networks using inducible transcription factors and transcriptome-wide time series. *Mol. Syst. Biol.* **16**, e9174 (2020).

27. K. A. Staschke, S. Dey, J. M. Zaborske, L. R. Palam, J. N. McClintick, T. Pan, H. J. Edenberg, R. C. Wek, Integration of general amino acid control and target of rapamycin (TOR) regulatory pathways in nitrogen assimilation in yeast. *J. Biol. Chem.* **285**, 16893–16911 (2010).

28. C. T. Harbison, D. B. Gordon, T. I. Lee, N. J. Rinaldi, K. D. Macisaac, T. W. Danford, N. M. Hannett, J.-B. Tagne, D. B. Reynolds, J. Yoo, E. G. Jennings, J. Zeitlinger, D. K. Pokholok, M. Kellis, P. A.

Rolfe, K. T. Takusagawa, E. S. Lander, D. K. Gifford, E. Fraenkel, R. A. Young, Transcriptional regulatory code of a eukaryotic genome. *Nature* **431**, 99–104 (2004).

29. T. Ben-Shitrit, N. Yosef, K. Shemesh, R. Sharan, E. Ruppin, M. Kupiec, Systematic identification of gene annotation errors in the widely used yeast mutation collections. *Nat. Methods* **9**, 373–378 (2012).

30. N. Atias, M. Kupiec, R. Sharan, Systematic identification and correction of annotation errors in the genetic interaction map of Saccharomyces cerevisiae. *Nucleic Acids Res.* **44**, e50 (2016).

31. A. A. Egorov, A. I. Alexandrov, V. N. Urakov, D. S. Makeeva, R. O. Edakin, A. S. Kushchenko, V. N. Gladyshev, I. V. Kulakovskiy, S. E. Dmitriev, A standard knockout procedure alters expression of adjacent loci at the translational level. *Nucleic Acids Res.* **49**, 11134–11144 (2021).

32. B. A. Cohen, R. D. Mitra, J. D. Hughes, G. M. Church, A computational analysis of whole-genome expression data reveals chromosomal domains of gene expression. *Nat. Genet.* **26**, 183–186 (2000).

33. B. Regenberg, T. Grotkjaer, O. Winther, A. Fausbøll, M. Akesson, C. Bro, L. K. Hansen, S. Brunak, J. Nielsen, Growth-rate regulated genes have profound impact on interpretation of transcriptome profiling in Saccharomyces cerevisiae. *Genome Biol.* **7**, R107 (2006).

34. S. C. Janga, J. Collado-Vides, M. M. Babu, Transcriptional regulation constrains the organization of genes on eukaryotic chromosomes. *Proc. Natl. Acad. Sci. U.S.A.* **105**, 15761–15766 (2008).

35. S. A. Teichmann, R. A. Veitia, Genes encoding subunits of stable complexes are clustered on the yeast chromosomes: An interpretation from a dosage balance perspective. *Genetics* **167**, 2121–2125 (2004).

36. J. M. Lee, E. L. L. Sonnhammer, Genomic gene clustering analysis of pathways in eukaryotes. *Genome Res.* **13**, 875–882 (2003).

37. M. A. Hibbs, D. C. Hess, C. L. Myers, C. Huttenhower, K. Li, O. G. Troyanskaya, Exploring the functional landscape of gene expression: Directed search of large microarray compendia. *Bioinformatics* **23**, 2692–2699 (2007).

38. J. M. Dempster, I. Boyle, F. Vazquez, D. E. Root, J. S. Boehm, W. C. Hahn, A. Tsherniak, J. M. McFarland, Chronos: A cell population dynamics model of CRISPR experiments that improves inference of gene fitness effects. *Genome Biol.* **22**, 343 (2021).

39. M. Wainberg, R. A. Kamber, A. Balsubramani, R. M. Meyers, N. Sinnott-Armstrong, D. Hornburg, L. Jiang, J. Chan, R. Jian, M. Gu, A. Shcherbina, M. M. Dubreuil, K. Spees, W. Meuleman, M. P. Snyder, M. C. Bassik, A. Kundaje, A genome-wide atlas of co-essential modules assigns function to uncharacterized genes. *Nat. Genet.* **53**, 638–649 (2021).

40. T. Wang, H. Yu, N. W. Hughes, B. Liu, A. Kendirli, K. Klein, W. W. Chen, E. S. Lander, D. M. Sabatini, Gene essentiality profiling reveals gene networks and synthetic lethal interactions with oncogenic ras. *Cell* **168**, 890–903.e15 (2017).

41. B. Rauscher, F. Heigwer, L. Henkel, T. Hielscher, O. Voloshanenko, M. Boutros, Toward an integrated map of genetic interactions in cancer cells. *Mol. Syst. Biol.* **14**, e7656 (2018).

42. E. R. McDonald III, A. de Weck, M. R. Schlabach, E. Billy, K. J. Mavrakis, G. R. Hoffman, D. Belur, D. Castelletti, E. Frias, K. Gampa, J. Golji, I. Kao, L. Li, P. Megel, T. A. Perkins, N. Ramadan, D. A. Ruddy, S. J. Silver, S. Sovath, M. Stump, O. Weber, R. Widmer, J. Yu, K. Yu, Y. Yue, D. Abramowski, E. Ackley, R. Barrett, J. Berger, J. L. Bernard, R. Billig, S. M. Brachmann, F. Buxton, R. Caothien, J. X. Caushi, F. S. Chung, M. Cortés-Cros, R. S. deBeaumont, C. Delaunay, A. Desplat, W. Duong, D. A. Dwoske, R. S. Eldridge, A. Farsidjani, F. Feng, J. Feng, D. Flemming, W. Forrester, G. G. Galli, Z. Gao, F. Gauter, V. Gibaja, K. Haas, M. Hattenberger, T. Hood, K. E. Hurov, Z. Jagani, M. Jenal, J. A. Johnson, M. D. Jones, A. Kapoor, J. Korn, J. Liu, Q. Liu, S. Liu, Y. Liu, A. T. Loo, K. J. Macchi, T. Martin, G. McAllister, A. Meyer, S. Mollé, R. A. Pagliarini, T. Phadke, B. Repko, T. Schouwey, F. Shanahan, Q. Shen, C. Stamm, C. Stephan, V. M. Stucke, R. Tiedt, M. Varadarajan, K. Venkatesan, A. C. Vitari, M. Wallroth, J. Weiler, J. Zhang, C. Mickanin, V. E. Myer, J. A. Porter, A. Lai, H. Bitter, E. Lees, N. Keen, A. Kauffmann, F. Stegmeier, F. Hofmann, T. Schmelzle, W. R. Sellers, Project DRIVE: A compendium of cancer dependencies and synthetic lethal relationships uncovered by large-scale, deep RNAi screening. *Cell* **170**, 577–592.e10 (2017).

43. J. Pan, R. M. Meyers, B. C. Michel, N. Mashtalir, A. E. Sizemore, J. N. Wells, S. H. Cassel, F. Vazquez, B. A. Weir, W. C. Hahn, J. A. Marsh, A. Tsherniak, C. Kadoch, Interrogation of mammalian

protein complex structure, function, and membership using genome-scale fitness screens. *Cell Syst.* **6**, 555–568.e7 (2018).

44. E. A. Boyle, J. K. Pritchard, W. J. Greenleaf, High-resolution mapping of cancer cell networks using co-functional interactions. *Mol. Syst. Biol.* **14**, e8594 (2018).

45. E. Kim, M. Dede, W. F. Lenoir, G. Wang, S. Srinivasan, M. Colic, T. Hart, A network of human functional gene interactions from knockout fitness screens in cancer cells. *Life Sci. Alliance* **2**, e201800278 (2019).

46. R. M. Meyers, J. G. Bryan, J. M. McFarland, B. A. Weir, A. E. Sizemore, H. Xu, N. V. Dharia, P. G. Montgomery, G. S. Cowley, S. Pantel, A. Goodale, Y. Lee, L. D. Ali, G. Jiang, R. Lubonja, W. F. Harrington, M. Strickland, T. Wu, D. C. Hawes, V. A. Zhivich, M. R. Wyatt, Z. Kalani, J. J. Chang, M. Okamoto, K. Stegmaier, T. R. Golub, J. S. Boehm, F. Vazquez, D. E. Root, W. C. Hahn, A. Tsherniak, Computational correction of copy number effect improves specificity of CRISPR-Cas9 essentiality screens in cancer cells. *Nat. Genet.* **49**, 1779–1784 (2017).

47. A. Baryshnikova, Data libraries—The missing element for modeling biological systems. *FEBS J.* **287**, 4594–4601 (2020).

48. M. D. Temple, A website to identify shared genes in Saccharomyces cerevisiae homozygous deletion library screens. *BMC Bioinformatics* **19**, 179 (2018).

49. S. G. Chuartzman, M. Schuldiner, Database for High Throughput Screening Hits (dHITS): A simple tool to retrieve gene specific phenotypes from systematic screens done in yeast. *Yeast* **35**, 477–483 (2018).

50. P. H. Thorpe, J. C. Dittmar, R. Rothstein, ScreenTroll: A searchable database to compare genome-wide yeast screens. *Database* **2012**, bas022 (2012).

51. Yeast Functional Genomics Database (YFGdb); http://yfgdb.princeton.edu/.

52. M. Galardini, B. P. Busby, C. Vieitez, A. S. Dunham, A. Typas, P. Beltrao, The impact of the genetic background on gene deletion phenotypes in Saccharomyces cerevisiae. *Mol. Syst. Biol.* **15**, e8831 (2019).

53. R. D. Dowell, O. Ryan, A. Jansen, D. Cheung, S. Agarwala, T. Danford, D. A. Bernstein, P. A. Rolfe, L. E. Heisler, B. Chin, C. Nislow, G. Giaever, P. C. Phillips, G. R. Fink, D. K. Gifford, C. Boone, Genotype to phenotype: A complex problem. *Science* **328**, 469 (2010).

54. S. Sun, A. M. Sendecki, S. Pullanchery, D. Huang, T. Yang, P. S. Cremer, Multistep interactions between ibuprofen and lipid membranes. *Langmuir* **34**, 10782–10792 (2018).

55. C. Laroche, L. Beney, P. A. Marechal, P. Gervais, The effect of osmotic pressure on the membrane fluidity of Saccharomyces cerevisiae at different physiological temperatures. *Appl. Microbiol. Biotechnol.* **56**, 249–254 (2001).

56. S. Barik, The uniqueness of tryptophan in biology: Properties, metabolism, interactions and localization in proteins. *Int. J. Mol. Sci.* **21**, 8776 (2020).

57. M. Kim, P. Tomek, Tryptophan: A rheostat of cancer immune escape mediated by immunosuppressive enzymes IDO1 and TDO. *Front. Immunol.* **12**, 636081 (2021).

58. M. J. Lercher, A. O. Urrutia, L. D. Hurst, Clustering of housekeeping genes provides a unified model of gene order in the human genome. *Nat. Genet.* **31**, 180–183 (2002).

59. C. Pál, L. D. Hurst, Evidence for co-evolution of gene order and recombination rate. *Nat. Genet.* **33**, 392–395 (2003).

60. M. Nei, Modification of linkage intensity by natural selection. *Genetics* **57**, 625–641 (1967).

61. D. Traxl, N. Boers, J. Kurths, Deep graphs—A general framework to represent and analyze heterogeneous complex systems across scales. *Chaos* **26**, 065303 (2016).

62. L. McInnes, J. Healy, J. Melville, UMAP: Uniform manifold approximation and projection for dimension reduction. arXiv:1802.03426 [stat.ML] (9 February 2018).

63. B. VanderSluis, D. C. Hess, C. Pesyna, E. W. Krumholz, T. Syed, B. Szappanos, C. Nislow, B. Papp, O. G. Troyanskaya, C. L. Myers, A. A. Caudy, Broad metabolic sensitivity profiling of a prototrophic yeast deletion collection. *Genome Biol.* **15**, R64 (2014).

64. M. S. Longtine, A. McKenzie III, D. J. Demarini, N. G. Shah, A. Wach, A. Brachat, P. Philippsen, J. R. Pringle, Additional modules for versatile and economical PCR-based gene deletion and modification in Saccharomyces cerevisiae. *Yeast* **14**, 953–961 (1998).

65. C. H. Ho, L. Magtanong, S. L. Barker, D. Gresham, S. Nishimura, P. Natarajan, J. L. Y. Koh, J. Porter, C. A. Gray, R. J. Andersen, G. Giaever, C. Nislow, B. Andrews, D. Botstein, T. R. Graham, M. Yoshida, C. Boone, A molecular barcoded yeast ORF library enables mode-of-action analysis of bioactive compounds. *Nat. Biotechnol.* **27**, 369–377 (2009).

66. E. Acton, A. H.-Y. Lee, P. J. Zhao, S. Flibotte, M. Neira, S. Sinha, J. Chiang, P. Flaherty, C. Nislow, G. Giaever, Comparative functional genomic screens of three yeast deletion collections reveal unexpected effects of genotype in response to diverse stress. *Open Biol.* **7**, 160330 (2017).

67. A. M. Deutschbauer, D. F. Jaramillo, M. Proctor, J. Kumm, M. E. Hillenmeyer, R. W. Davis, C. Nislow, G. Giaever, Mechanisms of haploinsufficiency revealed by genome-wide profiling in yeast. *Genetics* **169**, 1915–1925 (2005).

68. H. Cai, S. Kauffman, F. Naider, J. M. Becker, Genomewide screen reveals a wide regulatory network for di/tripeptide utilization in Saccharomyces cerevisiae. *Genetics* **172**, 1459–1476 (2006).

69. L. Kuepfer, U. Sauer, L. M. Blank, Metabolic functions of duplicate genes in Saccharomyces cerevisiae. *Genome Res.* **15**, 1421–1430 (2005).

70. F. Abe, H. Minegishi, Global screening of genes essential for growth in high-pressure and cold environments: Searching for basic adaptive strategies using a yeast deletion library. *Genetics* **178**, 851–872 (2008).

71. A. Y. Lee, R. P. St Onge, M. J. Proctor, I. M. Wallace, A. H. Nile, P. A. Spagnuolo, Y. Jitkova, M. Gronda, Y. Wu, M. K. Kim, K. Cheung-Ong, N. P. Torres, E. D. Spear, M. K. L. Han, U. Schlecht, S. Suresh, G. Duby, L. E. Heisler, A. Surendra, E. Fung, M. L. Urbanus, M. Gebbia, E. Lissina, M.

Miranda, J. H. Chiang, A. M. Aparicio, M. Zeghouf, R. W. Davis, J. Cherfils, M. Boutry, C. A. Kaiser, C. L. Cummins, W. S. Trimble, G. W. Brown, A. D. Schimmer, V. A. Bankaitis, C. Nislow, G. D. Bader, G. Giaever, Mapping the cellular response to small molecules using chemogenomic fitness signatures. *Science* **344**, 208–211 (2014).

72. E. Ericson, M. Gebbia, L. E. Heisler, J. Wildenhain, M. Tyers, G. Giaever, C. Nislow, Off-target effects of psychoactive drugs revealed by genome-wide assays in yeast. *PLOS Genet.* **4**, e1000151 (2008).

73. C. L. Tucker, S. Fields, Quantitative genome-wide analysis of yeast deletion strain sensitivities to oxidative and chemical stress. *Comp. Funct. Genomics* **5**, 216–224 (2004).

74. M. Mollapour, D. Fong, K. Balakrishnan, N. Harris, S. Thompson, C. Schüller, K. Kuchler, P. W. Piper, Screening the yeast deletant mutant collection for hypersensitivity and hyper-resistance to sorbate, a weak organic acid food preservative. *Yeast* **21**, 927–946 (2004).

75. C. Schüller, Y. M. Mamnun, M. Mollapour, G. Krapf, M. Schuster, B. E. Bauer, P. W. Piper, K. Kuchler, Global phenotypic analysis and transcriptional profiling defines the weak acid stress response regulon in Saccharomyces cerevisiae. *Mol. Biol. Cell* **15**, 706–720 (2004).

76. K. S. Dimmer, S. Fritz, F. Fuchs, M. Messerschmitt, N. Weinbach, W. Neupert, B. Westermann, Genetic basis of mitochondrial function and morphology in Saccharomyces cerevisiae. *Mol. Biol. Cell* **13**, 847–853 (2002).

77. L. M. Steinmetz, C. Scharfe, A. M. Deutschbauer, D. Mokranjac, Z. S. Herman, T. Jones, A. M. Chu, G. Giaever, H. Prokisch, P. J. Oefner, R. W. Davis, Systematic screen for human disease genes in yeast. *Nat. Genet.* **31**, 400–404 (2002).

78. A. M. Dudley, D. M. Janse, A. Tanay, R. Shamir, G. M. Church, A global view of pleiotropy and phenotypically derived gene function in yeast. *Mol. Syst. Biol.*, **1**, 2005.0001 (2005).

79. C. Luban, M. Beutel, U. Stahl, U. Schmidt, Systematic screening of nuclear encoded proteins involved in the splicing metabolism of group II introns in yeast mitochondria. *Gene* **354**, 72–79 (2005).

80. S. Merz, B. Westermann, Genome-wide deletion mutant analysis reveals genes required for respiratory growth, mitochondrial genome maintenance and mitochondrial protein synthesis in Saccharomyces cerevisiae. *Genome Biol.* **10**, R95 (2009).

81. W. Qian, D. Ma, C. Xiao, Z. Wang, J. Zhang, The genomic landscape and evolutionary resolution of antagonistic pleiotropy in yeast. *Cell Rep.* **2**, 1399–1410 (2012).

82. M. Stenger, D. T. Le, T. Klecker, B. Westermann, Systematic analysis of nuclear gene function in respiratory growth and expression of the mitochondrial genome in S. cerevisiae. *Microb. Cell Fact.* **7**, 234–249 (2020).

83. X. Teng, M. Dayhoff-Brannigan, W.-C. Cheng, C. E. Gilbert, C. N. Sing, N. L. Diny, S. J. Wheelan, M. J. Dunham, J. D. Boeke, F. J. Pineda, J. M. Hardwick, Genome-wide consequences of deleting any single gene. *Mol. Cell* **52**, 485–494 (2013).

84. J. van Leeuwen, C. Pons, J. C. Mellor, T. N. Yamaguchi, H. Friesen, J. Koschwanez, M. M. Ušaj, M. Pechlaner, M. Takar, M. Ušaj, B. VanderSluis, K. Andrusiak, P. Bansal, A. Baryshnikova, C. E. Boone, J. Cao, A. Cote, M. Gebbia, G. Horecka, I. Horecka, E. Kuzmin, N. Legro, W. Liang, N. van Lieshout, M. McNee, B.-J. San Luis, F. Shaeri, E. Shuteriqi, S. Sun, L. Yang, J.-Y. Youn, M. Yuen, M. Costanzo, A.-C. Gingras, P. Aloy, C. Oostenbrink, A. Murray, T. R. Graham, C. L. Myers, B. J. Andrews, F. P. Roth, C. Boone, Exploring genetic suppression interactions on a global scale. *Science* **354**, eaag0839 (2016).

85. R. Oughtred, J. Rust, C. Chang, B.-J. Breitkreutz, C. Stark, A. Willems, L. Boucher, G. Leung, N. Kolas, F. Zhang, S. Dolma, J. Coulombe-Huntington, A. Chatr-Aryamontri, K. Dolinski, M. Tyers, The BioGRID database: A comprehensive biomedical resource of curated protein, genetic, and chemical interactions. *Protein Sci.* **30**, 187–200 (2021).

86. S. Balaji, M. M. Babu, L. M. Iyer, N. M. Luscombe, L. Aravind, Comprehensive analysis of combinatorial regulation using the transcriptional regulatory network of yeast. *J. Mol. Biol.* **360**, 213–227 (2006).

87. C. L. Myers, D. R. Barrett, M. A. Hibbs, C. Huttenhower, O. G. Troyanskaya, Finding function: Evaluation methods for functional genomic data. *BMC Genomics* **7**, 187 (2006).

88. K. P. Byrne, K. H. Wolfe, The Yeast Gene Order Browser: Combining curated homology and syntenic context reveals gene fate in polyploid species. *Genome Res.* **15**, 1456–1461 (2005).

89. Alliance of Genome Resources Consortium, Harmonizing model organism data in the Alliance of Genome Resources. *Genetics* **220**, iyac022 (2022).

90. A. W. Hightower, W. A. Orenstein, S. M. Martin, Recommendations for the use of Taylor series confidence intervals for estimates of vaccine efficacy. *Bull. World Health Organ.* **66**, 99–105 (1988).

91. M. Mattiazzi Usaj, N. Sahin, H. Friesen, C. Pons, M. Usaj, M. P. D. Masinas, E. Shuteriqi, A. Shkurin, P. Aloy, Q. Morris, C. Boone, B. J. Andrews, Systematic genetics and single-cell imaging reveal widespread morphological pleiotropy and cell-to-cell variability. *Mol. Syst. Biol.* **16**, e9243 (2020).

92. V. Dederer, A. Khmelinskii, A. G. Huhn, V. Okreglak, M. Knop, M. K. Lemberg, Cooperation of mitochondrial and ER factors in quality control of tail-anchored proteins. *eLife* **8**, e45506 (2019).

93. D. Yu, J. Danku, I. Baxter, S. Kim, O. K. Vatamaniuk, D. E. Salt, O. Vitek, Noise reduction in genome-wide perturbation screens using linear mixed-effect models. *Bioinformatics* **27**, 2173–2180 (2011).

94. M. Matecic, D. L. Smith, X. Pan, N. Maqani, S. Bekiranov, J. D. Boeke, J. S. Smith, A microarray-based genetic screen for yeast chronological aging factors. *PLOS Genet.* **6**, e1000921 (2010).

95. W. A. Wilson, Z. Wang, P. J. Roach, Systematic identification of the genes affecting glycogen storage in the yeast Saccharomyces cerevisiae: Implication of the vacuole as a determinant of glycogen level. *Mol. Cell. Proteomics* **1**, 232–242 (2002).

96. S. H. Loukin, C. Kung, Y. Saimi, Lipid perturbations sensitize osmotic down-shock activated Ca^{2+} influx, a yeast “deletome” analysis. *FASEB J.* **21**, 1813–1820 (2007).
